# Computational optical sectioning with an incoherent multiscale scattering model for light-field microscopy

**DOI:** 10.1038/s41467-021-26730-w

**Published:** 2021-11-04

**Authors:** Yi Zhang, Zhi Lu, Jiamin Wu, Xing Lin, Dong Jiang, Yeyi Cai, Jiachen Xie, Yuling Wang, Tianyi Zhu, Xiangyang Ji, Qionghai Dai

**Affiliations:** 1 Department of Automation, Tsinghua University, 100084 Beijing, China; 2 Institute for Brain and Cognitive Sciences, Tsinghua University, 100084 Beijing, China; 3 Beijing National Research Center for Information Science and Technology, Tsinghua University, 100084 Beijing, China; 4 State Key Laboratory of Membrane Biology, Tsinghua University–Peking University Joint Centre for Life Sciences, Beijing Frontier Research Centre for Biological Structure, School of Life Sciences, Tsinghua University, 100084 Beijing, China

**Keywords:** Data processing, 3-D reconstruction, Ca2+ imaging, Fluorescence imaging

## Abstract

Quantitative volumetric fluorescence imaging at high speed across a long term is vital to understand various cellular and subcellular behaviors in living organisms. Light-field microscopy provides a compact computational solution by imaging the entire volume in a tomographic way, while facing severe degradation in scattering tissue or densely-labelled samples. To address this problem, we propose an incoherent multiscale scattering model in a complete space for quantitative 3D reconstruction in complicated environments, which is called computational optical sectioning. Without the requirement of any hardware modifications, our method can be generally applied to different light-field schemes with reduction in background fluorescence, reconstruction artifacts, and computational costs, facilitating more practical applications of LFM in a broad community. We validate the superior performance by imaging various biological dynamics in Drosophila embryos, zebrafish larvae, and mice.

## Abstract (web summary)

Light-field microscopy provides volumetric imaging at high speeds, but suffers from degradation in scattering tissue. Here, the authors present an incoherent multiscale scattering model which allows for quantitative 3D reconstruction in complex environments, and demonstrate dynamic imaging in vivo.

## Introduction

The beauty of life lies in the complexity and variety of cellular behaviors in three-dimensional (3D) living organisms, which are difficult to appreciate without advanced imaging techniques^1–3^. To meet the increasingly urgent need, various methods have been developed in the past decade, including high-speed scanning^4–6^, point spread function (PSF) engineering^7,8^, and multiplexing several discrete focal planes^9,10^. Among them, light-field microscopy (LFM)^11^, as an emerging computational imaging framework featuring with high photon efficiency, data throughput, and system compactness, has shown its unique advantages in various applications including imaging hemodynamics^12^, large-scale neural activities^13,14^ and long-term intercellular and intracellular interactions^15,16^. Different from traditional imaging with a shallow depth of field (DOF)^9,10,17^, LFM captures the entire 3D volume in a tomographic manner within the extended depth of field and maximize the data parallelization, leading to orders-of-magnitude reduction in phototoxicity^15^. However, as a wide-field-esque imaging method, LFM still suffers from great degradation and loss of quantitative properties with the existence of scattering^18^ or dense fluorescence labeling^19^, restricting its broad applications in deep tissue such as mammalian brains and embryos.

To address this problem, speckle illumination^20^, light-sheet illumination^12^, and confocal schematics^21^ have been proposed recently for additional optical sectioning. Despite the enhanced signal-to-background ratio (SBR), these approaches either require sample priors^20^, space constraints on sample geometry^12^ or complicated hardware modifications^12,21^. Moreover, the resolutions of these methods are usually much lower than the diffraction limit of whole objective numerical aperture (NA) due to the tradeoff between spatial and angular resolutions. By exploring the diffraction of the microlens at the image plane, scanning LFM (sLFM) can bypass the tradeoff and permit long-term high-speed subcellular imaging in mammals^15^, but it’s hard to apply confocal schematics in sLFM, restricting its performance in densely labeled samples.

Here, we propose a computationally efficient, incoherent multiscale multiple-scattering model for all kinds of LFM to achieve computational optical sectioning in densely labeled or scattering samples without the requirement of any hardware modifications. To distinguish from traditional LFM, this method is termed as quantitative LFM (QLFM). We find that the severe degradation of traditional LFM in thick tissue mainly results from the incomplete space and ideal imaging models used during reconstruction. By considering various factors together, including the nonuniform resolution of different axial planes, 3D fluorescence across a large range, multiple scattering, and system aberrations, we can not only improve the resolution and contrast with reduced computational costs but also push LFM to the quantitative level with alleviated crosstalk and artifacts in complicated environments, which is critical for various quantitative biological analysis. In addition, we show QLFM is compatible with different schematics of LFM such as unfocused LFM and scanning LFM (sLFM). To demonstrate the versatile applications of QLFM, especially in densely labeled and scattering samples, we imaged various biological dynamics in different specimens, including zebrafish larvae heart beating, blood flow, and whole-brain neural activity, *Drosophila* embryo development, and mouse brain neural activities. Our multiscale multiple-scattering model allowed us to achieve high-fidelity 3D fluorescence data with computational optical sectioning, notably better than that of traditional ideal models, while maintaining system compactness.

## Results

### Principle and implementation of QLFM

**Fig. 1:**
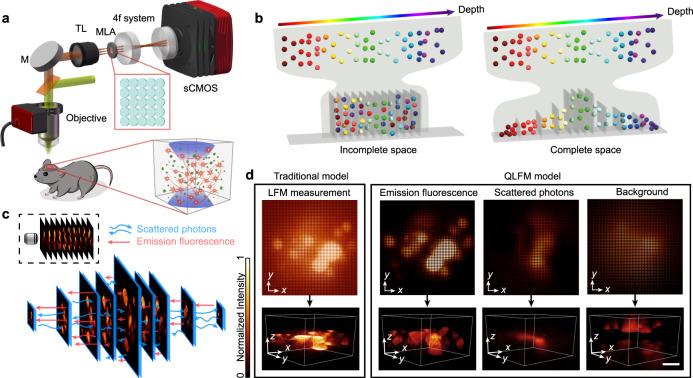
Schematic of quantitative light-field microscopy (QLFM). **a** Experimental setup of our QLFM system with a simple microlens array (MLA) inserted at the image plane for snapshot phase-space measurements. M mirror, TL tube lens, sCMOS (scientific complementary metal-oxide semiconductor) camera. **b** Concept of the multiscale model. Incomplete space used in the reconstruction of traditional LFM results in strong background noises and loss of quantitative properties in complicated environments. We apply different sampling rates in 3D based on the effective resolution of LFM at different axial planes to model a large volume for background rejection with an orders-of-magnitude reduction in computational costs. **c** Schematic of the multiscale scattering model. We differentiate the multiple-scattered photons from native emission fluorescence in the multislice model based on the first Born approximation to retrieve 3D fluorescence quantitatively in deep tissue. **d** Comparisons between traditional LFM and QLFM by imaging the same GFP-labeled B16 tumor spheroid. With a more accurate physics model to describe the imaging process, QLFM can separate the spatially nonuniform background and scattered photons from native 3D emission fluorescence, facilitating computational optical sectioning. Scale bars, 30 μm.

As a general computational model for incoherent conditions, our QLFM method is compatible with different schematics of LFM. Here, we chose the unfocused LFM for most experimental demonstrations by inserting a microlens array at the image plane of a normal wide-field epifluorescence microscope (Fig. 1a). A 4f system was used to relay the back focal plane of the microlens array at the image sensor, with each microlens covering ~13 × 13 sensor pixels (Methods section). With the matched NA, LFM provides an extended depth of field much larger than that of WFM^9,10,17^. The fluorescence signals far from the focal plane exhibit similar Gaussian backgrounds in WFM, while they show apparent distinguishable features between different angular components in LFM within a snapshot (Supplementary Fig. 1 and Supplementary Movie 1). We find that these depth-dependent features provide LFM with a similar capability of computational optical sectioning as structured illumination microscopy^22^. The 3D out-of-focus fluorescence up to the centimeter scale can be reconstructed at low resolution, as long as we take it into consideration in the multislice model as a complete space. These layers far from the native objective plane were usually neglected in previous methods due to the limited computational resources and contributed considerable background noises and artifacts in the high-resolution range (Fig. 1b). To address this problem, we propose a multiscale model in the phase-space domain by resampling the volume based on the nonuniform resolution of different axial planes, avoiding many unnecessary calculations in a complete space (Fig. 1b, Supplementary Fig. 2, and Methods section). We then conducted a numerical simulation with gradually increasing background levels to show the improved quantitative property of the multiscale model, which is barely considered in previous studies but important for biological analysis (Supplementary Fig. 3). The reconstructed volumes in traditional LFM are distorted without modeling of the nonuniform 3D background, while QLFM shows much better robustness.

Except for out-of-focus fluorescence, the scattered photons pose another challenge in thick tissue, which cannot be rejected by traditional optical sectioning due to the depth-independent property. Although multislice scattering models have achieved great success in the coherent imaging modalities^23^, they are barely considered in deconvolution algorithms for incoherent fluorescence imaging. As the 4D phase-space measurements by LFM can fully describe the partially coherent light-field distributions, they provide an opportunity to infer the native 3D fluorescence as well as the nonuniform 3D scattering coefficients. Here, we derived a multislice multiple-scattering model to differentiate the emission fluorescence and scattered photons based on the first Born approximation in the incoherent condition^24^ (Fig. 1c and Supplementary Note 1). Then, we applied the alternating direction method of multipliers (ADMM) algorithm^25^ with the multiscale model to update the volumetric fluorescence and 3D scattering potentials iteratively (Supplementary Fig. 2 and Supplementary Movie 1).

By modeling both the out-of-focus and scattered photons, we retrieved the 3D fluorescence distribution quantitatively in thick tissue. To show the pipeline, we imaged a GFP-labeled tumor spheroid with a ×63/1.4 NA oil-immersion objective. Strong nonuniform background fluorescence and scattered photons were observed in the traditional LFM, which were effectively removed by the capability of computational optical sectioning in QLFM (Fig. 1d). In addition, since the high-resolution 3D range of LFM is fixed with a specific objective, axial scanning at a large step is usually required for large-volume samples in practice to make full use of the surplus 3D imaging speed. However, we find that the background-induced artifacts in traditional LFM lead to the failure of volume stitching in the axial domain (Supplementary Fig. 4). The axial side, with larger out-of-focus photons, tends to have higher intensities, demonstrating the loss of the quantitative property in thick tissue. As shown in Supplementary Fig. 4, QLFM can eliminate these background artifacts and facilitate continuous volume reconstructions with uniform resolution, as long as we take the entire 3D volume as a whole with the multiscale sampling rate during reconstruction (Methods section).

### Characterization of QLFM

**Fig. 2:**
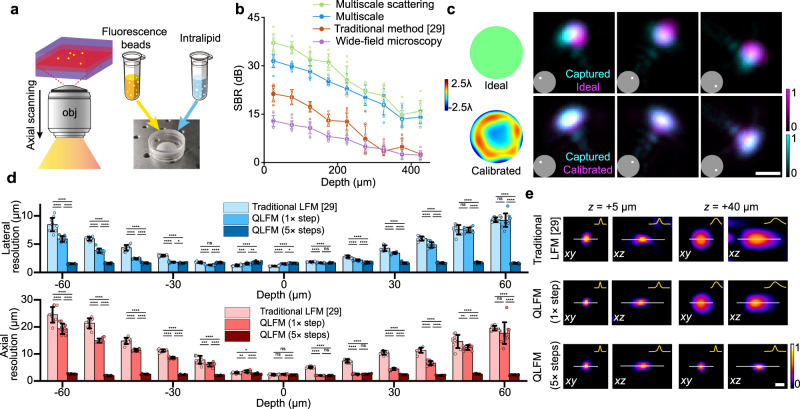
Characterization of quantitative light-field microscopy (QLFM). **a** Illustrations of the SBR characterization experiment. We fabricated the scattering phantom with the mixture of 0.025% 2-μm fluorescence beads, 1% intralipid, and 1% agarose in a petri dish. Then we imaged the sample with a 40×/1.0NA water-immersion objective (Obj) at different penetration depths with WFM and LFM. **b** SBR curves of the fluorescence beads at different penetration depths in the intralipid-based tissue-mimicking phantom obtained by WFM, traditional LFM, QLFM with the multiscale model only, and QLFM with the multiscale scattering model. Data are presented as means ± SD. We chose 10 fluorescence beads with the highest fitting degrees for each block covering ~40 μm. QLFM shows ~20 dB improvement in the SBR over WFM, indicating an improved penetration depth in deep tissue. **c** Comparisons among the experimental PSF, the ideal PSF without aberrations, and the calibrated PSF of several selected angular components marked in the inset under the ×40/1.0 NA water-immersion objective. The calibrated wavefront of the system aberration estimated by our iterative phase-retrieval algorithm is shown on the left. **d** Lateral and axial resolution at different axial planes in traditional LFM and QLFM with 1 and 5 axially scanned steps, which is estimated by the FWHM of subdiffraction-limited fluorescence beads. Bar graphs are represented as means ± SD. For each block covering 10 μm, we chose 10 fluorescence beads with the highest fitting degrees for statistical analysis (one-way ANOVA with Tukey’s multiple comparisons test; the significance threshold was set at *α* = 0.05). *p < 0.05, **p < 0.01, ***p < 0.001, ****p < 0.0001, ns not significant. **e** Reconstructed orthogonal slices of 0.5-μm-diameter fluorescence beads located at depths of 5 μm and 40 μm away from the focal plane by traditional LFM, QLFM with and without axial scanning of 5 steps. Cross-section profiles along the marked lines were plotted in the insets. Source data are provided as a Source data file. Scale bar: 3 μm.

To quantitatively evaluate the improvement of QLFM, we imaged various 2-μm fluorescence beads embedded in a tissue-mimicking phantom made of intralipid and agarose with a ×40/1.0 NA objective and calculated the average SBR at different penetration depths (Methods section, Fig. 2a and Supplementary Fig. 5). By comparing with WFM and different LFM models (Fig. 2b), we found that the naive imaging model of traditional LFM resulted in a similar performance as WFM in tissue penetration. Our multiscale scattering model fully exploits the capability of LFM in deep-tissue imaging with ~20 dB SBR improvement over WFM. Such a great improvement facilitates QLFM high-speed 3D imaging in thick tissue. We then imaged the same densely labeled *Drosophila* brain by QLFM, traditional LFM, WFM, and confocal microscopy for comparison (Supplementary Fig. 6). Despite reduced resolution due to the tradeoff between spatial and angular resolutions in LFM, QLFM showed great sectioning capability without axial-scanning artifacts, which was much better than those of traditional LFM and WFM.

Another problem hindering the quantitative reconstruction in LFM is the inaccurate estimation of the complicated high-dimensional PSF. As shown in Fig. 2c, the system aberration will result in great system errors between the ideal PSF and the experimental PSF measured with a subdiffraction-limited fluorescence bead. We propose a phase-retrieval^26,27^ based algorithm to estimate the system aberration by iteratively shrinking the disparity between the simulated PSF and the captured PSF along different angular components with a single image (Supplementary Fig. 7). We find that the calibrated PSF with the aberration wavefront can greatly reduce the reconstruction artifacts close to the native objective plane (Supplementary Fig. 7c). Numerical simulations on different levels of aberrations also show the effectiveness of the method (Supplementary Fig. 8). We then characterized the system resolution by imaging 500-nm fluorescence beads distributed in 1% agarose with a ×40/1.0 NA water-immersion objective. The average full width at half-maximum (FWHM) was used to estimate the lateral and axial resolutions at different axial planes (Fig. 2d, e). The calibrated PSF improves the spatial resolution, especially in axial domain. Moreover, QLFM with high-speed axial scanning at a step size of 30 μm for five planes has a uniform resolution of ~1.8 × 1.8 × 2.5 μm^3^ across a large depth range covering ~330 × 330 × 180 μm^3^.

As the multiscale model in phase-space domain avoids the unnecessary up-sampling based on the effective resolution, it can also reduce the memory and computing time by orders of magnitude, especially for large depth ranges during reconstruction to get rid of background. By modeling the same volume range, our multiscale model with adaptive sampling rate shows the same performance as traditional methods, but reduces the computing time from several hours to several seconds (Supplementary Fig. 9), on a desktop computer equipped with a normal graphical processing unit (CPU: Intel i9-9980XE, RAM: 128 GB, GPU: NVIDIA GeForce RTX 2080 Ti). Such reduction in computational costs is crucial for practical and broad applications of LFM in biology with the great generalization ability.

### High-speed 3D imaging of *Drosophila* embryo and Zebrafish larvae

**Fig. 3:**
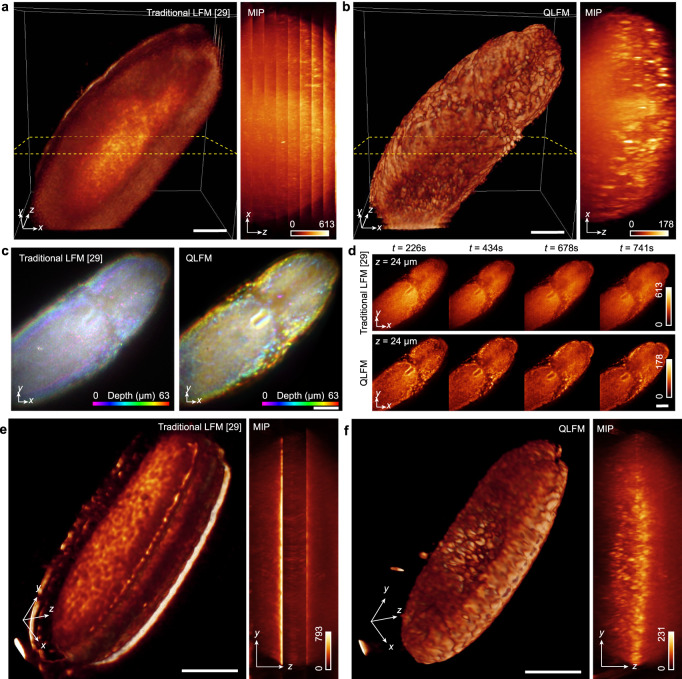
Experimental comparisons on the histone-labeled *Drosophila* embryo with high-speed axial scanning. **a**, **b** 3D rendered volumes and 180-μm-*xz* maximum intensity projections (MIP) at the same time point obtained by traditional LFM and QLFM with a 40×/1.0NA water-immersion objective at an axial step of 15 μm. QLFM shows much better contrast and resolution than traditional LFM without artifacts, illustrating the capability of computational optical sectioning by QLFM with the improved penetration depth. **c** Depth-coded MIP of a *Drosophila* embryo reconstructed by traditional LFM and QLFM. **d** Slices reconstructed by different methods at various time stamps. **e**, **f** The comparisons between traditional LFM and QLFM under a ×20/0.5NA objective in the form of 3D rendered volumes and orthogonal MIPs with axial scanning at a step of 50 μm, indicating the uniform resolution of QLFM without edge artifacts. The images were processed with Volren modules in Amira 5.4 to get the volume renderings with similar contrast. Scale bars, 50 μm (**a–d**) and 100 μm (**e**, **f**).

To demonstrate the superior performance of QLFM over previous methods using compact systems, we performed in vivo imaging of various fast biological dynamics in different specimens. First, we imaged the whole-embryo development of histone-labeled *Drosophila* at the millisecond scale with both ×40/1.0 NA (Fig. 3a–d) and ×20/0.5 NA (Fig. 3e, f) objectives for comparisons. Different from previous methods capturing only part of the volume at a time^28^, LFM achieves better photon efficiency with low phototoxicity by illuminating and detecting the entire volume simultaneously^15^. While traditional LFM^29^ mistook the scattering and background photons as the effective information without an accurate model, leading to larger maximum intensities and lower contrasts, QLFM could still distinguish single cells deep in the embryo at the millisecond scale, extending the applications of LFM to developmental biology (Fig. 3a–d). Such an improvement in contrast cannot be achieved by simple background rejection, as the in-focus information will be flooded by the nonuniform background signals (Supplementary Fig. 10). Especially for the axial domain, traditional LFM has severe artifacts during volume stitching, which is hard to localize single cells (Fig. 3e, f). In addition, while previous methods highly rely on the sparse prior in spatiotemporal domain of neuron firing to reduce the background^30,31^, QLFM is suitable for long-term imaging of 3D morphological changes without the requirement of the sparse prior.

**Fig. 4:**
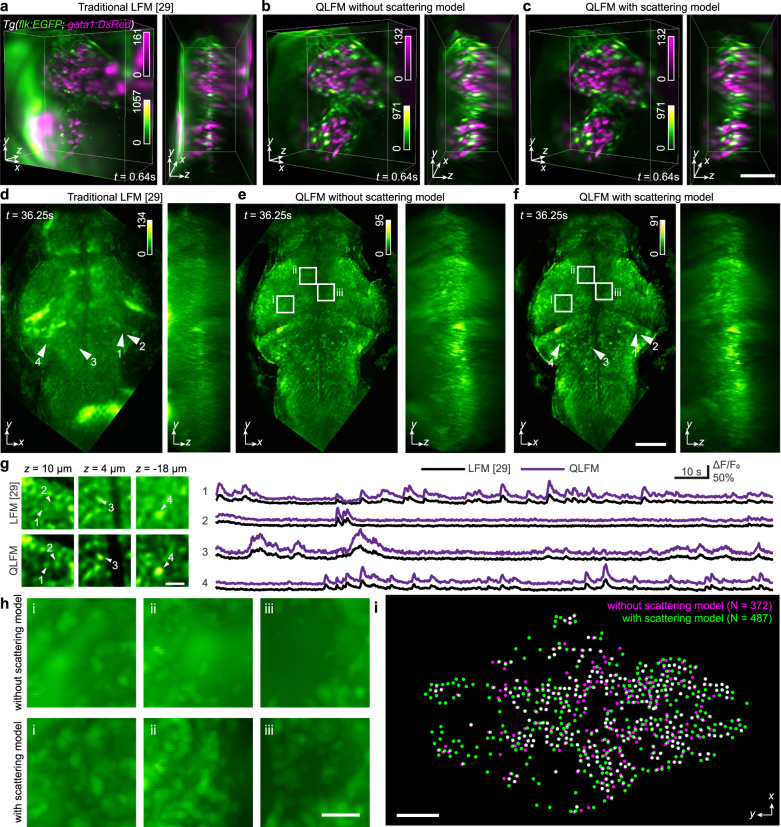
High-speed volumetric imaging in larval zebrafish. **a–c** 3D rendered volumes of the beating heart at the same time point reconstructed by the traditional LFM, QLFM without the scattering model, and QLFM with the scattering model (Supplementary Movie 2). Successive improvements can be observed with reduced artifacts and background for quantitative imaging. **d–f** Orthogonal MIPs of the whole brain at the same time point reconstructed by the traditional LFM, QLFM without the scattering model, and QLFM with the scattering model, illustrating the effectiveness of computational optical sectioning in densely labeled samples (Supplementary Movie 3). **g** Zoom-in regions of the same neurons marked in **d** and **f** and their temporal traces for comparison, illustrating the improved SBR. **h** The white-box regions in **e** and **f** are zoomed-in to show that neurons flooded by the scattered photons can be resolved with our multiscale scattering model. **i** Segmentation results (QLFM without the scattering model, magenta; QLFM with the scattering model, green; overlap, white) of 2000 frames reconstructed by different methods, indicating that more neurons can be resolved with the scattering model. Scale bars, 50 μm (**a–c**), 100 μm (**d–f**), and 20 μm (**g**, **h**).

Then, we performed in vivo imaging of heart-beating dynamics in zebrafish larvae, which are difficult to fully capture without simultaneous exposure of the whole volume at high-speed. Traditional LFM shows low resolution and contrast with severe artifacts at both the center and edge (Fig. 4a). On the contrary, QLFM, with a more accurate computational model, could obtain artifact-free high-resolution 3D volumes without the requirement of additional light-sheet illumination and multiview objectives (Fig. 4b, c and Supplementary Fig. 11). We found successive improvement with increasing model complexity, indicating the necessity of various factors in the model, including out-of-focus fluorescence, sample scattering, and system aberrations (Supplementary Fig. 11 and Supplementary Movie 2). Such an improvement in contrast and reduction in artifacts are very important for the downstream applications such as large-scale cell tracking and cell segmentations (Supplementary Figs. 11 and 12).

Whole-brain neural recording in zebrafish larvae is another typical application of LFM, but the imaging performance is far from satisfactory due to the densely packed neurons without complicated systems^14^ (Fig. 4d). Although the spatiotemporal sparsity facilitates the accurate localization and extraction of neural signals^30,31^, it is still susceptible to nonuniform background fluorescence and inevitable structural changes in living animals. By imaging the whole-brain neural activities in zebrafish larvae at 24 Hz with a ×20/0.5 NA objective, we demonstrate that QLFM can achieve effective single-neuron resolution with proper models to remove both out-of-focus and scattered photons for each single frame without the requirement to calculate the standard deviation of many frames for better contrast (Fig. 4e, f, and Supplementary Movie 3). The temporal traces of several neurons with simple region-of-interest (ROI) averaging demonstrate the increased contrast and reduced crosstalk between adjacent neurons in QLFM (Fig. 4g). To show the effectiveness of the scattering model, we further compared ROIs and found more neurons resolved by reducing the nonuniform scattered photons (Fig. 4h, i).

### Large-scale 3D calcium imaging in mice brain

**Fig. 5:**
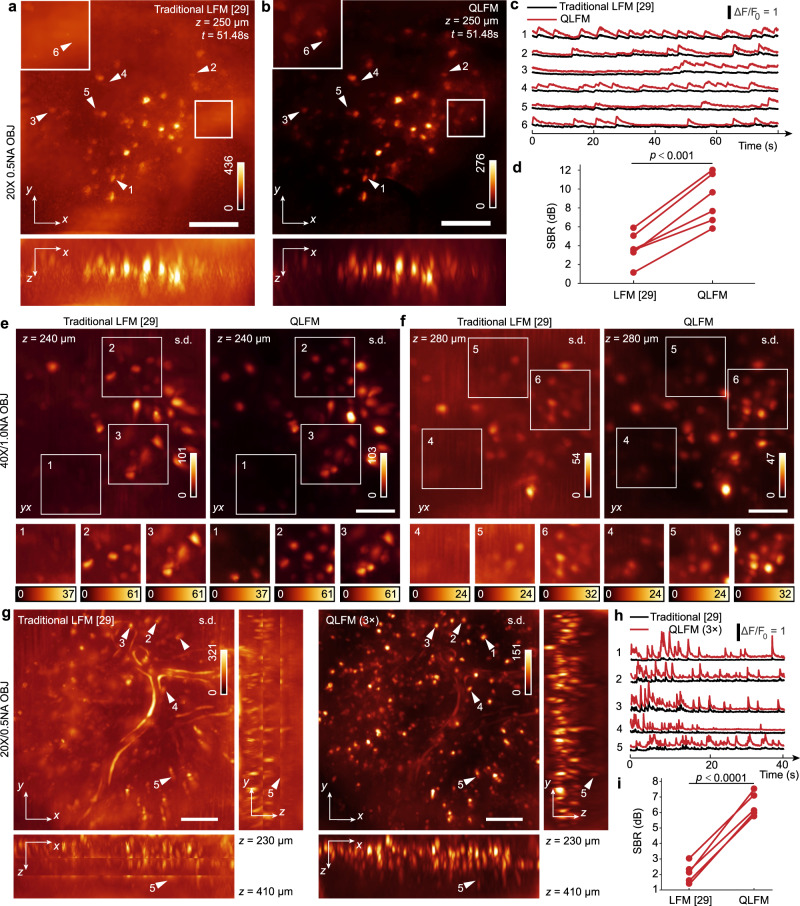
3D functional imaging in awake mouse brains. **a**, **b** Orthogonal MIPs of GCaMP6s-labeled neurons at *t* = 51.48 s reconstructed by traditional LFM and QLFM with a ×20/0.5NA objective, indicating the reduced background with computational optical sectioning (Supplementary Movie 4). More neurons were revealed by QLFM in the mouse cortex with the native objective plane at a depth of ~250 μm. **c** Temporal traces of the marked neurons in **a** and **b**, demonstrating the quantitative calcium responses in QLFM with improved contrast. **d** Scatter diagram of SBR of each neuron marked in **a** and **b** by different methods. Data points from the same neuron obtained by traditional LFM and QLFM are connected by a red line. *P* value was calculated by the paired, two-tailed *t*-test (*α* = 0.05). **e**, **f** Orthogonal MIPs of the standard deviation across 2000 volumes imaged at a center depth of 240 μm and 280 μm in the cortex, respectively, under a ×40/1.0 NA water-immersion objective (Supplementary Movie 5). **g** Orthogonal MIPs of the standard deviation across 500 volumes for GCaMP6f-labeled L2/3 neurons in awake behaving mice. The video was captured with high-speed axial scanning at a step of 50 μm for three planes under a ×20/0.5 NA objective. **h** Temporal traces of several marked neurons at different depths. **i** Scatter diagram of SBR for five selected neurons in **g**. Data points from the same neuron obtained by traditional LFM and QLFM are connected by a red line. *P* value was determined via the paired, two-tailed *t*-test (*α* = 0.05). Source data are provided as a Source data file. Scale bars, 100 μm (**a**, **b**, **g**) and 50 μm (**e**, **f**).

The mammalian brain is a more challenging case due to its strong scattering and dense neural structures. We tested the performance of QLFM by imaging the 3D calcium activities in an awake head-fixed mouse, which was labeled with GCaMP6s by virus injection, under a ×20/0.5 NA objective. To visualize the 3D distribution of neurons, traditional LFM usually needs to calculate the standard deviation of thousands of frames to reduce the background (Supplementary Fig. 13a). However, the 3D signals of a single frame are usually flooded in the background fluctuations due to the low SBR (Fig. 5a). In contrast, with the capability of computational optical sectioning, QLFM shows significantly improved contrast (Fig. 5b and Supplementary Movie 4) and the fluorescence changes ΔF/F_0_ is consistent with previous studies^32^. In addition to the detection of more neurons (Supplementary Fig. 13a), QLFM allows the measurement of calcium dynamics in a quantitative manner, making it more reliable for large-scale neural recording (Fig. 5c). To quantitatively analyze the improvement of SBR, we estimated the background from the average of the same small regions without neurons in different methods and the signal using the average of the temporal traces of the selected neuron somas. As the firing of the neuron is rare, the calculated SBR is relatively small. However, SBR increase can still be observed in QLFM (Fig. 5d). Background fluorescence usually increases with the increasing of NA due to the smaller depth of field, especially for the wide-field imaging. We therefore imaged the same mouse with a 40×/1.0 NA water-immersion objective at different depths (Fig. 5e, f, Supplementary Fig. 13b–e, and Supplementary Movie 5). QLFM resolved more neurons with better resolution and contrast than traditional LFM, and showed stable performance at different depths up to 300 μm (Fig. 5e, f). In addition, we show that such improvement cannot be obtained by simply subtracting the background in traditional LFM (Supplementary Fig. 13f). To quantitatively analyze the improvement, a direct CNMF segmentation^33^ was applied to the MIP of the 1000 volumes reconstructed by different methods (Supplementary Fig. 14a). Results show that more neurons can be extracted in QLFM. The Pearson correlation between the neurons and the corresponding background is significantly suppressed, indicating the reduced crosstalk from scattered and background photons (Supplementary Fig. 14b).

By high-speed axial scanning, we could further increase the axial coverage with redundant 3D imaging speed for calcium dynamics. We then conducted millisecond-scale calcium imaging in awake double-transgenic Rasgrf2-2A-dCre/Ai148D mice across a large volume covering ~700 × 700 × 180 μm^3^ by high-speed scanning of three planes. While there was barely contrast in traditional LFM due to the severe artifacts especially in the axial domain, which was similar to previous work^30^, QLFM showed a much larger penetration depth with uniform resolution (Fig. 5g and Supplementary Movie 6). Even the neurons located ~400 μm deep exhibited remarkable calcium responses in QLFM (Fig. 5g, h). The significant increasement of SBR also suggests the reduced crosstalk and effective improvement. With computational optical sectioning, QLFM has the potential to facilitate more quantitative downstream analysis in various biological applications (Fig. 5h, i).

## Discussion

In summary, we developed an incoherent multiscale scattering model to fully exploit the intrinsic high-dimensional property of light-field measurements and achieved computational optical sectioning, facilitating high-speed, large-scale, quantitative 3D imaging in deep tissue without the requirement of any hardware modifications. Our method can effectively remove different kinds of reconstruction artifacts in LFM, originated from system aberrations and severe crosstalk from nonuniform background fluorescence and scattered photons. We conducted various experiments on *Drosophila* embryo, zebrafish larvae, and awaking mice to show the capability of high-speed morphological and functional 3D imaging at single cell resolution in densely labeled scattering samples with computational optical sectioning. Quantitative evaluations on fluorescence beads in tissue-mimic phantom and neural activities in mice cortex show at least 3-fold improvement in SBR by QLFM, illustrating its high fidelity for large-scale 3D neural recording in scattering tissue without any sparse priors.

Different from wide-field imaging with small depth of field, light-field imaging provides a tomographic framework by keeping the photons focused across a large depth range along different angles. Such a process makes the signals originated from different axial planes show much more distinguishable features than WFM, which can be utilized to remove background fluorescence far from the native objective plane computationally, as long as we model the imaging process in a complete space. Additional confocal rejection or light-sheet illuminations will definitely further reduce the shot-noise fluctuations by getting rid of the background photons physically but at the cost of system compactness, space constraints, or detection parallelization.

In addition, as a computational model, our method is compatible with different light-field imaging schemes without the requirement of any hardware modifications. While current QLFM still suffers from the low spatial resolution due to the tradeoff between spatial and angular resolutions, scanning LFM^15^ can be combined with QLFM for the long-term observation of subcellular dynamics in densely labeled samples (Supplementary Fig. 15). Despite nonuniform background rejection, the axial resolution of QLFM at the low spatial-frequency range is still lower than confocal microscopy due to the missing-cone problem, which may be addressed by structured illuminations in the future. In any case, the incoherent multiscale scattering model provides the orders-of-magnitude reductions in background fluorescence, artifacts, and computational costs, enabling the practical and versatile applications of QLFM in the broad biology community as a compact and robust add-on to normal wide-field microscopy.

## Methods

### Experimental setup

Our QLFM system works as an add-on to a normal epifluorescence microscope, with a microlens array inserted at the image plane. A customized upright microscope was used for mouse experiments, while a commercial inverted microscope (Zeiss Observer Z1) was used for the others. Another 4f system (with a magnification of 0.845) relayed the back focal plane of the microlens array (with a pitch size of 100 μm and focal length of 2.1 mm) to the camera (Andor Zyla 4.2 plus, 2048 × 2048 pixels) so that each microlens covered ~13 × 13 sensor pixels, corresponding to a 2.15 μm × 2.15 μm area at the sample plane for the ×40/1.0 NA water-immersion objective. Andor Solis (ver3.40) software was used for data collection. Labview 2018 was used for hardware synchronization. Multiple lasers (488 nm and 561 nm) were used for the fluorescence excitation of multiple channels, which were synchronized with the camera for time division multiplexing. A piezo objective scanner (PI P-725.4CD) was used for high-speed axial scanning with a resolution of 1.25 nm at 100 Hz. Detailed imaging conditions and reconstruction parameters for all fluorescence experiments in the paper, including the excitation power, exposure time, frame rate, voxel size, fluorophore, protein, filter set, and objective, are illustrated in Supplementary Table 1.

### 3D deconvolution with a multiscale scattering model

To reconstruct the 3D fluorescence information in deep tissue in a quantitative way, we propose a 3D deconvolution algorithm with a multiscale scattering model to iteratively update both the emission fluorescence and scattering photons across a large depth range (Fig. 1c). The whole pipeline of the algorithm with the pseudocode is shown in Supplementary Fig. 2.

With simple pixel realignment, the raw LF data can be represented as multiple angular components, or smoothed phase-space measurements^34^, which can be used for phase-space deconvolution^29^ to reduce artifacts and increase the convergence speed. However, the reconstructed volume, regardless of the deconvolution algorithm^29,35^, is limited to only dozens of axial planes due to the heavy computational cost. Therefore, the out-of-focus fluorescence has usually been modeled as a constant in previous methods, which is fine for thin samples but leads to severe artifacts and background noise in deep tissue with nonuniform out-of-focus distributions. Fortunately, both the lateral and axial resolution of LFM will gradually decrease with increasing distances from the native objective plane. We can establish a multiscale grid to sample a large 3D volume at different intervals based on the characterized resolution with an exponential fit (Supplementary Fig. 2). The out-of-focus fluorescence with a nonuniform 3D distribution can then be estimated with the depth-dependent features within a large depth of field (Supplementary Fig. 1), akin to structured illumination microscopy exhibiting computational optical sectioning. As this method does not require dense axial sampling and a lateral sampling rate as high as the camera pixel number for each plane, which was necessary in previously reported methods due to the spatially variant PSF^35^, the unknown variables can be reduced by orders of magnitude to accelerate the reconstruction speed when modeling the out-of-focus fluorescence of a much larger volume (Supplementary Fig. 9). For the same volume size, our multiscale model can achieve almost the same performance as traditional dense-sampling models at a much faster speed. In contrast, the traditional method^29^, without a sufficient axial range in the model, shows apparent artifacts and an increase in the background.

Scattering is a recurring challenge in thick tissue and is the fundamental limitation of the penetration depth in light microscopy, leading to reduced resolution and contrast^36^. Although the multilayer scattering model has recently achieved great success in coherent imaging modalities, such as diffraction tomography^37^, the spatially nonuniform influence of scattering is barely considered in fluorescence imaging. LFM provides an opportunity to differentiate emission fluorescence and scattered photons with its 4D phase-space measurements. We propose a multilayer scattering model based on the first Born approximation in incoherent conditions by describing the relation between the emission fluorescence ${I}^{(i)}({{{{{\bf{r}}}}}})$ and scattered photons ${I}^{(s)}({{{{{\bf{r}}}}}})$ as follows:

1 $${I}^{(s)}({{{{{\bf{r}}}}}})=\frac{1}{2} \int_{V}F({{{{{\bf{r}}}}}}^{\prime}){I}^{(i)}({{{{{\bf{r}}}}}}^{\prime})\frac{1}{{\Vert {{{{{\bf{r}}}}}}-{{{{{\bf{r}}}}}}^{\prime}\Vert }_{2}^{2}}{{{{{{\rm{d}}}}}}}^{3}r^{\prime}$$

where *F* is defined as the scattering potential energy at the 3D position *r*, and *v* is the whole 3D volume range. The detailed derivations and discretized version are illustrated in Supplementary Note 1. Then, an ADMM framework^25^ is used to iteratively update the sample information and the scattered photons to retrieve the quantitative fluorescence distributions in deep tissue with increased contrast and resolution (Supplementary Fig. 2). The reconstruction time of a single volume with a full FOV of the sensor used here is about several seconds for a normal desktop computer with a graphical processing unit.

### 3D deconvolution with axial scanning for LFM

The high-resolution 3D range of LFM for a snapshot is fixed for a specific objective. High-speed axial scanning at a large step size is a straightforward method to flexibly adjust the depth range and 3D imaging speed. Such a capability is important for versatile applications with different requirements for the volume size and imaging speed. However, traditional methods show severe artifacts at the stitching edges at low contrast (Figs. 3a and 5g and Supplementary Fig. 4), as they usually reconstruct each subvolume based on every single LF image separately and stitch the subvolumes in the axial domain^30^. Here, with the multiscale model in QLFM, we take the entire 3D volume as a whole with the multiscale sampling rate during reconstruction. As shown in Supplementary Movie 1, after pixel realignment of all the raw LF images, we can achieve multiple focal stacks for different angular components, making full use of the intrinsic continuous property of different angular components in the axial domain. Due to the large depth of field for each angular component in QLFM, we can use a large step for axial scanning with much less time required to cover the same volume than in WFM. Then we update the entire large volume as a whole along different angles, viewing each angular focal stack as the minimum unit to calculate the error map for each iteration. Such a process is akin to wide-field deconvolution first for each angle followed by tomographic reconstruction later for different angles. Finally, the same ADMM framework is applied to the entire volume to update the emission fluorescence and scattered photons iteratively. In this case, uniform resolution can be achieved at different axial planes across a large depth range without any artifacts (Fig. 2d and Supplementary Fig. 4). In addition, the computational cost can be further reduced, as we can conduct 3D deconvolution with the phase-space PSF for each angular focal stack. For continuous volume reconstruction with uniform resolution, the axial step size should be similar to the high-resolution axial range of LFM, which is determined by the numerical aperture (NA) of the objective and the angular resolution. In our experiments, we set the step size as 10 and 50 μm respectively for ×63/1.4 NA and ×20/0.5 NA.

### Phase-retrieval-based PSF calibration

The PSF of traditional LFM is calculated based on wave optics theory in an ideal imaging condition^29,35^. However, the experimental imaging system usually has complicated system aberrations, which will not only reduce the imaging resolution but also introduce severe reconstruction artifacts close to the native objective plane. Here, we propose a phase-retrieval-based algorithm to calibrate the experimental PSF with a single image of subdiffraction-limited fluorescence beads (Supplementary Fig. 7). The simulated PSF is first initialized with experimental parameters without any aberration wavefront. Then, we calculate the correlations between the captured image and the simulated PSF along different angular components to estimate the wavefront error at different sub-apertures. The aberrated wavefront of the whole NA is then integrated from the correlation map for continuous distributions. The estimated wavefront is fitted with Zernike polynomials and fed into the wave optics model to generate the new simulated PSF, which is used again to match with the captured PSF. The calibrated PSF is iteratively updated through the above process until the phase map converges, which usually takes ~3–4 iterations. Interestingly, we found that the experimental system with spherical aberrations can remove the reconstruction artifacts close to the native objective plane as long as we had an accurate estimation of the aberration wavefront.

### Fluorescence bead preparation for system characterization

For resolution characterization, 500-nm yellow-green fluorescent microspheres (Thermo Scientific, FluoSpheres, carboxylate-modified microspheres) were mixed with 1% agarose at a ratio of 1:1,000,000. We placed the mixture in a 35-mm petri dish and captured ~100 LF images for statistical analysis with a 40×/1.0 NA water-immersion objective. We then calculated the FWHM of the lateral and axial profiles of the reconstructed beads on different axial planes. For each block covering 10 μm, we chose 10 beads with the highest fitting degree to calculate the mean and standard deviation.

For SBR characterization, we fabricated a scattering phantom with the mixture of 1% agarose, 1% intralipid (Absin 68890-65-3, 20% emulsion), and 0.025% 2-μm fluorescence beads, which was placed in a 35-mm petri dish. The 2-μm fluorescence beads (Thermo Scientific, FluoSpheres, carboxylate-modified microspheres) were randomly distributed in the intralipid and imaged using a ×40/1.0 NA water-immersion objective. The reconstructed mean intensity of the beads was viewed as the signal, while the mean intensity of the reconstructed background without samples was viewed as the background. Several reconstructed slices at different imaging depths are shown in Supplementary Fig. 5.

### Tumor spheroid preparation

B16 cells (ATCC® CRL-6475™, mouse skin melanoma cells) were purchased from ATCC and cultured in RPMI 1640 medium supplemented with 10% FBS, 1% pen/strep antibiotics, and 1% NEAA (all from GIBCO). Cells were then transfected with the EGFP-PH plasmid (Addgene Plasmid #96948), and stable EGFP-expressing B16 cells were selected by neomycin (G418) and maintained. To prepare tumor spheroids, 4 × 10^3^ EGFP-expressing B16 cells per well were seeded in round-bottomed 96-well plates (Corning) and cultured in RPMI 1640 medium supplemented with 10% FBS, 2% B-27 supplement (GIBCO), 2% methyl cellulose (Sigma-Aldrich), 1% pen/strep antibiotics, and 1% NEAA. After 2 days, each formed spheroid was transferred as one spheroid per well and cultured for another 2 days. During LFM imaging, the GFP-B16 tumor spheroids were transferred to Lab-Tek II cover-glass-bottomed 8-chamber slides and imaged in HBSS supplemented with 2% FBS (all from Invitrogen) using a 63×/1.4 NA oil-immersion objective.

### In vivo imaging of zebrafish larvae

All zebrafish experimental procedures were conducted with ethical approval from the Animal Care and Use Committee of Tsinghua University. For live imaging of embryo cells, zebrafish embryos were injected with 300 pg *Tspan4a*-EGFP mRNA (synthesized in vitro with mMessage mMachine T7 kit, Ambion, AM1344) in one cell at 16-cell stage. During imaging, zebrafish embryos were embedded in 1% agarose in 35-mm confocal dishes (Cellvis, D35-10-0-N) and the *Tspan4*-EGFP expressing embryo cells were imaged at 70% epiboly stage. For imaging of the vasculature and blood flow dynamics, Tg(*flk:EGFP*; *gata1:DsRed*) transgenic zebrafish embryos were collected and cultured at 28.5 °C in Holtfreter’s solution. At 4–5 days postfertilization (dpf), the zebrafish larvae were anesthetized by ethyl 3-aminobenzoate methanesulfonate salt (100 mg/L) and mounted in 1% low-melting-point agarose for imaging at 26–27 °C. For whole-brain calcium imaging, Tg(*huc:GCaMP6*) transgenic zebrafish embryos were collected and kept at 28.5 °C in Holtfreter’s solution. At 4–5 dpf, the larvae were mounted in 1% low-melting-point agarose for imaging at 26–27 °C.

### Preparation of fixed *Drosophila* brain samples

All *Drosophila* experimental procedures were conducted with ethical approval from the Animal Care and Use Committee of Tsinghua University. Female *Drosophila* brains were dissected and fixed in 4% paraformaldehyde (PFA, Cat# AR-0211, Dingguo Biotech, China) at room temperature for 30 mins on a shaker. Each brain was rehydrated with 0.3% Triton X-100 (Solarbio 524A0513) in phosphate-buffered saline (PBS) for 4 × 20 mins at room temperature and then incubated in block solution (5% goat serum in washing buffer) for 30 mins at room temperature. The brain was then incubated overnight with primary antibody (mouse monoclonal nc82 (Developmental Studies Hybridoma Bank)), which was diluted at 1:500 in block solution at 4 °C. The brain was then washed in 0.5% PBST for 3 × 1 h at room temperature. Finally, the brain was mounted directly for imaging by LFM.

### Imaging of *Drosophila* embryos

All *Drosophila* embryos (Fig. 3 and Supplementary Fig. 10) expressed histones tagged with EGFP (His2Av, BL24163). The collection and preparation of *Drosophila* embryos were performed according to the commonly used protocol^38^. Two-hour *Drosophila* embryos were collected within a specific collection chamber. After incubation at 25 °C for 10 h, each embryo was attached to a glass microscope slide with double-sided sticky tape. We used forceps to carefully roll the embryo on the tape until its chorion popped open. Then, the embryo was transferred to the glues line on a dish (Ibidi µ-Dish, 35 mm, high) and covered with mineral oil (Sigma-Aldrich, Halocarbon 27 oil) for live imaging.

### Mouse experiments

All procedures involving mice were approved by the Institutional Animal Care and Use Committee of Tsinghua University. We used both male and female C57BL/6 mice 10 weeks to 6 months old without randomization or blinding. Mice were group-housed under a cycle of 12 h light/dark (lights on at 7 a.m.) and provided with water and food ad libitum. The relative humidity was 50% at 20–22 °C. We performed the craniotomy^39^ with a window size of an 8-mm diameter. Briefly, mice were anesthetized with 2% isoflurane and fixed on the stereotaxic apparatus (RWD, China). The scalp was removed by sterile surgical scissors to expose the entire dorsal skull. The skull was thoroughly cleaned with saline to remove all fascia above skull. Then, a piece of skull (8 mm in diameter) was removed using cranial drill and replaced with a crystal skull. The edge of the crystal skull and the skin incision were sealed with cyanoacrylate (Vetbond, 3 M). A custom-made head-post was implanted above the skull and fixed with dental cement. After surgery, the mice were injected with flunixin meglumine (1.25 mg/kg) for 5 consecutive days. For acute imaging, we used adult double-transgenic Rasgrf2-2A-dCre/Ai148D mice (JAX No.: 022864 and 030328) to specifically label cortical layer 2/3 neurons^40^. For chronic imaging, adult C57BL/6 mice injected with diluted AAV9-hSyn-GCaMP6s virus (from BrainVTA Technology Co., Ltd., China) were allowed to recover for at least 2 weeks after craniotomy. During LFM imaging, awake mice were placed in a tube with the head restrained under the objective.

### Ethics statement

This project complied with all relevant ethical regulations for animal testing and research. All experimental procedures were conducted with ethical approval from the Animal Care and Use Committee of Tsinghua University.

### Data analysis

All data analyses were performed with customized MATLAB (MathWorks, MATLAB 2018) programs and Amira (Thermo Fisher Scientific, Amira 2019). The hardware was controlled using LabVIEW 2018 (National Instruments). The data was collected by Andor Solis (ver3.40). The 3D rendering of the volumes in figures and videos was performed by Amira. Details of the parameters and models we used are listed in Supplementary Table 2. The 3D tracking of 7 representative blood cells in the heart of the zebrafish larvae was carried out manually in MATLAB.

### Neural activity extraction

The calcium responses in both zebrafish and mice were extracted directly through signal averaging of the manually selected ROIs covering the selected neurons. The ROIs were ~8 × 8 × 10 μm^3^ in size for zebrafish larvae and ~10 × 10 × 10 μm^3^ in size for mice to match the size of the neuron. The temporal traces of neural activity were calculated by *ΔF*/*F*_*0*_ = (*F- F*_*0*_)/*F*_*0*_, where *F* is the raw averaged intensity of the ROI, and *F*_*0*_ is the baseline fluorescence intensity. To estimate *F*_*0*_ for each ROI, we first calculated the average intensity of the trace and averaged all time points with signals below 120% of the calculated average. The SBR was calculated as the ratio between time-average of signal (average intensity of selected ROIs) and time-average of background (average intensity of areas without neurons) in Fig. 5d, i. For fair comparisons, we chose the same regions in different methods.

### Performance metrics

We used Pearson correlation to illustrate that QLFM is capable of reducing the background cross-talks. The Pearson correlation is calculated by the following formula:

2 $$r(X,Y)=\frac{E[(X-\bar{X})(Y-\bar{Y})]}{{\sigma }_{X}{\sigma }_{Y}}$$

where *X* and *Y* are signals, $\bar{X}$ and $\bar{Y}$ are mean values of each signal, ${\sigma }_{X}$ and ${\sigma }_{Y}$ are the corresponding standard deviations.

### Statistics and reproducibility

Experiments in Fig. 1d and Supplementary Fig. 4 are repeated on 12 frames, showing similar performance. Data shown in Figs. 2, 3 and Supplementary Figs. 3, 5, 6, 7, 8, 10, and 15 are representative of *n* = 5 experiments. Data shown in Fig. 4, and Supplementary Figs. 9, 11, and 12 are representative of *n* = 10 experiments. Data shown in Fig. 5 and Supplementary Fig. 13 are representative of *n* = 12 experiments.

### Reporting summary

Further information on research design is available in the Nature Research Reporting Summary linked to this article.

## Supplementary information

Supplementary Information

Description of Additional Supplementary Files

Supplementary Movie 1

Supplementary Movie 2

Supplementary Movie 3

Supplementary Movie 4

Supplementary Movie 5

Supplementary Movie 6

Reporting Summary

## Data Availability

The data generated in this study has been made publicly available at https://github.com/yizhang-zww18/QLFM_dataset Source data are provided with this paper.

## Source data

Source Data

## Code availability

The code for QLFM is available at 10.5281/zenodo.5524512^41^ and https://github.com/yizhang-zww18/QLFM.
