# Supplementary material for: Computational optical sectioning with an incoherent multiscale scattering model for light-field microscopy
Source: Nat Commun. 2021 Nov 4;12:6391. doi: 10.1038/s41467-021-26730-w (PMC8568979; doi:10.1038/s41467-021-26730-w)
Supplement: Supplementary file 1 — Supplementary Information [file 41467_2021_26730_MOESM1_ESM.pdf]

Supplementary Information

Computational optical sectioning with an incoherent multiscale scattering model for light-field microscopy

Yi Zhang[1,2*], Zhi Lu[1,2*], Jiamin Wu[1,2*,†], Xing Lin[1,2,3], Dong Jiang[4], Yeyi Cai[1], Jiachen Xie[1,2], Yuling Wang[1], Tianyi Zhu[1,2], Xiangyang Ji[1,2†] & Qionghai Dai[1,2,3†]

[1]Department of Automation, Tsinghua University, Beijing, 100084, China

[2]Institute for Brain and Cognitive Sciences, Tsinghua University, Beijing 100084, China,

[3]Beijing National Research Center for Information Science and Technology, Tsinghua University, Beijing 100084, China

[4]State Key Laboratory of Membrane Biology, Tsinghua University–Peking University Joint Centre for Life Sciences, Beijing Frontier Research Centre for Biological Structure, School of Life Sciences, Tsinghua University, Beijing, 100084, China.

[*]These authors contributed equally to this work

[†]Correspondence: wujiamin@tsinghua.edu.cn (J. W.), xyji@tsinghua.edu.cn (X. J.), qhdai@tsinghua.edu.cn (Q. D.)

**Supplementary Figure 1**	Illustrations of the extended depth of field (DOF) by light-field imaging for background modeling
**Supplementary Figure 2**	The pseudo codes (left) and corresponding illustrations (right) of 3D deconvolution with a multiscale scattering model
**Supplementary Figure 3**	Numerical analysis of the quantitative property of QLFM and traditional LFM with increasing background levels
**Supplementary Figure 4**	Large-scale volumetric imaging of GFP-labeled tumor spheroids with axial scanning.
**Supplementary Figure 5**	Characterization of the signal-to-background ratio (SBR) in the tissue-mimicking phantom
**Supplementary Figure 6**	Experimental comparisons among confocal microscopy, WFM, traditional LFM, and QLFM on the same fixed *Drosophila* brain
**Supplementary Figure 7**	The pseudo codes and corresponding illustrations for phase-retrieval-based PSF calibration
**Supplementary Figure 8**	Quantitative analysis of the phase-retrieval-based PSF calibration algorithm
**Supplementary Figure 9**	Illustrations of the effectiveness of multiscale model to reduce the memory and computing time by orders of magnitude
**Supplementary Figure 10**	Experimental comparisons on *Drosophila* embryo between traditional LFM and QLFM
**Supplementary Figure 11**	Experimental comparisons on the beating heart in zebrafish larvae to show improvements
**Supplementary Figure 12**	Experimental comparisons on the large-scale blood flow in zebrafish larvae between traditional LFM and QLFM
**Supplementary Figure 13**	In vivo 3D calcium imaging in mouse cortex
**Supplementary Figure 14**	Comparisons on the performance of subsequent data analysis between traditional LFM and QLFM

**Supplementary Figure 15**	Supplementary Figure 15 | Experimental comparisons on the zebrafish embryo with scanning LFM
**Supplementary Note 1**	Incoherent scattering model for 3D deconvolution
**Supplementary Table 1**	Imaging conditions for all fluorescence experiments
**Supplementary Table 2**	Rendering parameters and models for all fluorescence experiments.
**Supplementary Movie 1**	Concept and pipeline of the multiscale scattering model for 3D deconvolution
**Supplementary Movie 2**	Heart-beating dynamics in zebrafish larvae imaged at 25 Hz by two channels to show the improvements with the model complexity
**Supplementary Movie 3**	Experimental comparisons on whole-brain calcium imaging of zebrafish larvae at 24Hz
**Supplementary Movie 4**	Experimental comparisons on 3D calcium imaging of a virus-injected awake mouse with a 20×/0.5 NA objective
**Supplementary Movie 5**	Experimental comparisons on 3D calcium imaging of a virus-injected awake mouse with a 40×/1.0 NA water-immersion objective
**Supplementary Movie 6**	Experimental comparisons on 3D calcium imaging of a transgenic awake mouse with high-speed axial scanning

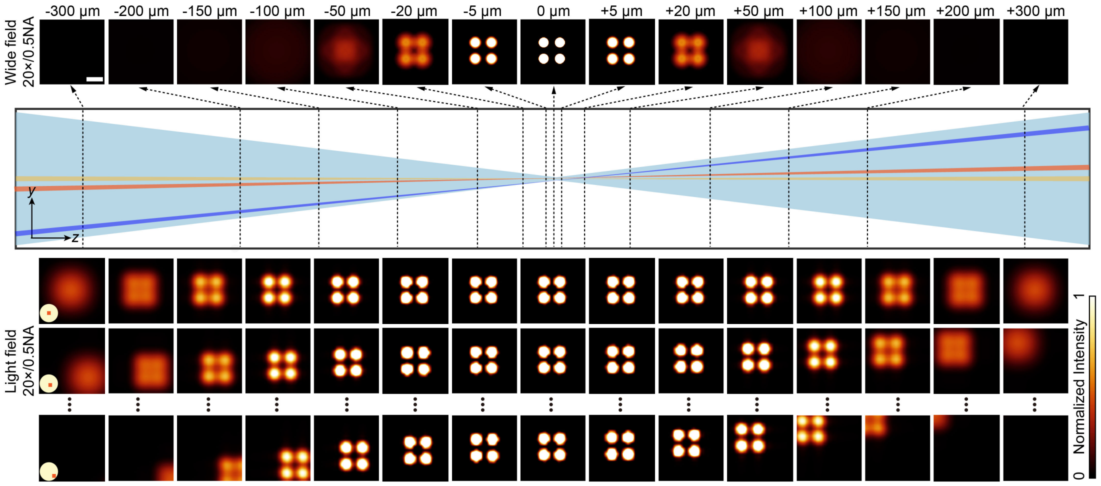

Supplementary Figure 1 | Illustrations of the extended depth of field (DOF) by light-field imaging for background modeling. We show the simulated images of four 8-μm fluorescence beads placed at different axial positions imaged with a 20×/0.5NA objective by wide-field microscopy (WFM) and light-field microscopy (LFM), respectively. The LF images of specific angular components after pixel realignment are shown for comparisons. The photons disperse quickly in WFM with the increase of the out-of-focus distances, generating a nonuniform background, which is hard to be removed without contrast. On the contrary, the photons in LFM are kept focused within an extended DOF and have unique depth-dependent shifts in different angular components, which can be easily rejected once modeled in the algorithm. Scale bar, 20 μm.

Algorithm 3D deconvolution with multiscale scattering model

Input: point spread function $H(\rho,u,nz)$, axial downsample rate r_z, lateral downsample rate r_{xy}, Spatial-angular components $W(\rho,u)$, maximum iteration M, initial volume $X_{all}(\rho,nz)$, initial scattering volume $I^{(s)}(\rho,nz)$, Green function G, update rate λ, ratio β, learning rate α
Output: descattered 3D volume $X(\rho,z)$

```
H(ρ,u,nz) = DS_xy(DS_z(H(ρ,u,nz),r_z),r_xy)
X_all(ρ,nz) = DS_xy(DS_z(X_all(ρ,nz),r_z),r_xy)
I^(s)(ρ,nz) = DS_xy(DS_z(I^(s)(ρ,nz),r_z),r_xy)

for m = 1:M do
    for u = 1:size(H,3) do
        if (m==1 && u ==1) do
            X(ρ,nz) = RL_deconv(H_u(ρ,nz), W_u(ρ), X(ρ,nz), λ)
                // Update X(ρ,nz) along u with RL deconvolution
            X_all(ρ,nz) = X(ρ,nz)
        end if
        X_all(ρ,nz) = RL_deconv(H_u(ρ,nz), W_u(ρ), X_all(ρ,nz), λ)
            // Update X_all(ρ,nz) along u with RL deconvolution
        L = ||FP(X_all(ρ,nz),u)-W_u(ρ))||^2
        for l = 1:size(X,3) do
            I^(i)(ρ,lz) = I^(i)(ρ,(l-1)z)**G + I^(i)(ρ,(l+1)z)**G
            ∂L/∂F(l) = [2(FP(X_all(ρ,nz),u)-W_u(ρ))**H_u^T(ρ,nz)]
                                .* I^(i)(ρ,nz)
            // ** convolution operator
            // .* point multiplication operator
            F(l) = F(l) - α·∂L/∂F(l)
        end for
        // Update F using gradient descent algorithm
        for l = 1:size(X(,3) do
            I^(s)(ρ,lz) = I^(i)(ρ,lz) .* F(l)
        end for
        X(ρ,nz)  =  X_all(ρ,nz) - I^(s)(ρ,nz)
            // Update X(ρ,nz) based on X_all(ρ,nz)
    end for
end for
X(ρ,nz) = UP_xy(UP_z(X(ρ,nz),r_z),r_xy)
Return X(ρ,nz)
```

Algorithm RL_deconv

Input: point spread function of a specific spatial-angular component $H_u(\rho,nz)$, specific spatial-angular component $W_u(\rho)$, initial volume $X(\rho,nz)$, update rate λ
Output: updated 3D volume *Xguess*

```
HX(ρ) = FP(X(ρ,nz),u)
HXBack(ρ,nz) = BP(HX(ρ),u)
IBack(ρ,nz) = BP(W_u(ρ),u)
ErrorBack(ρ,nz) = IBack ./ HXBack
                    //  ./ point division operator
X(ρ,nz) = λ*X(ρ,nz)+(1-λ)*ErrorBack(ρ,nz)
Return X(ρ,nz)
```

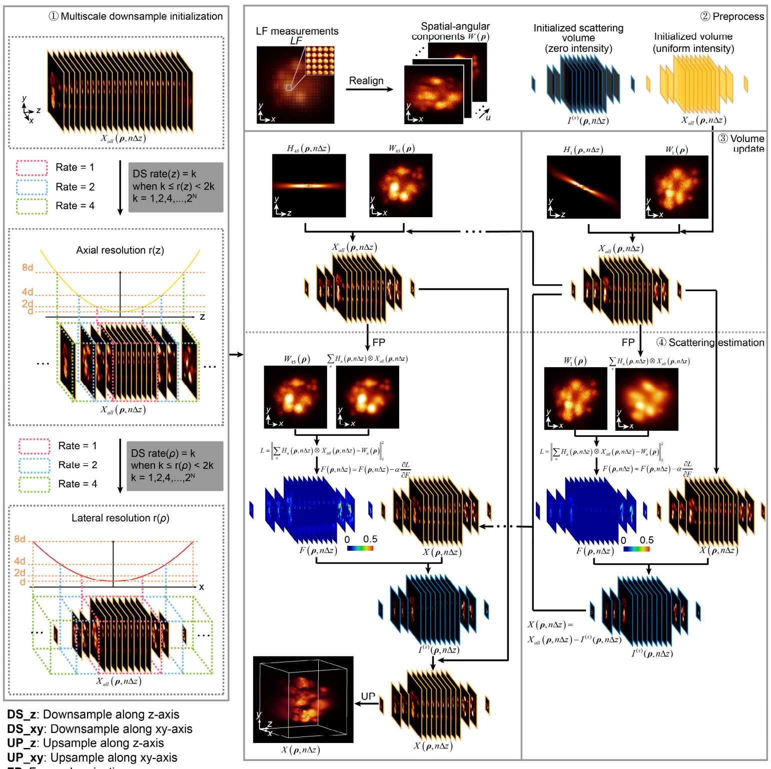

DS_z: Downsample along z-axis
DS_xy: Downsample along xy-axis
UP_z: Upsample along z-axis
UP_xy: Upsample along xy-axis
FP: Forward projection
BP: Backward projection

Supplementary Figure 2 | The pseudo codes (left) and corresponding illustrations (right) of 3D deconvolution with a multiscale scattering model. In traditional LF models, the volume initialized for reconstruction is densely sampled with a uniform sampling rate, which is very redundant due to the nonuniform low resolution of LFM at different axial planes. Consequently, only the high-resolution 3D range close to the native objective plane is modeled in traditional LFM to save the computational cost. On the contrary, our multiscale model considers a much larger volume to update the 3D nonuniform background fluorescence together with the high-resolution 3D range during reconstruction. Both the volume and point spread functions (PSF) are down-sampled with different sampling rates according to the characterized resolutions at different axial planes, resulting in orders-of-magnitude reduction in computational cost without resolution degradation. Then an ADMM framework is used to update the native 3D volume and scattered potentials iteratively. Only a specific angular component is used within each iteration to accelerate the convergence.

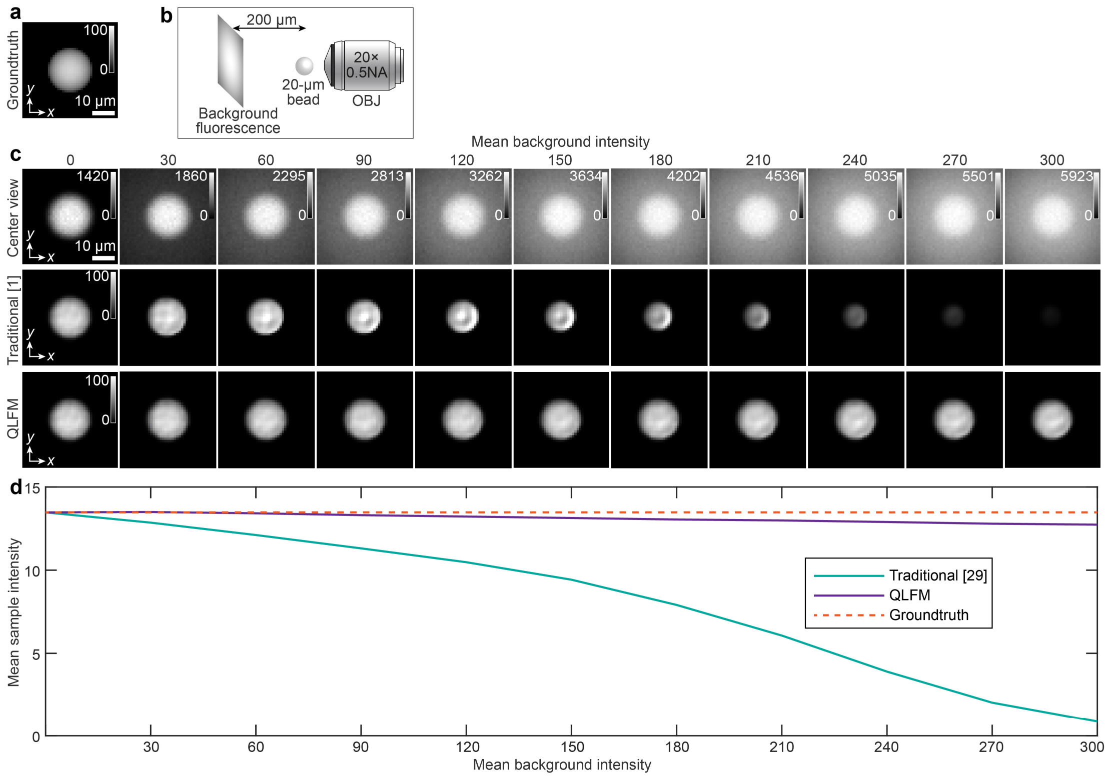

Supplementary Figure 3 | Numerical analysis of the quantitative property of QLFM and traditional LFM with increasing background levels. a, A simulated 20-μm-diameter fluorescence bead is placed at the focal plane as groundtruth with 100 photons in each voxel covering 0.33×0.33×0.5 μm^3. **b**, The simulated imaging schematic with a Gaussian-distributed background located at 200 μm away from the native objective plane. Both the background and the bead are imaged under the 20×/0.5NA objective (OBJ). **c**, The simulated center views with shot noise and 42-μm mean intensity projections reconstructed by traditional LFM[1] and QLFM, respectively, with different mean background intensities marked on the top. With the increase of the background, the structural information and the intensity of the bead will gradually decrease in traditional LFM due to the crosstalk from the background fluorescence, illustrating the loss of the quantitative property in thick tissue. On the contrary, QLFM show significantly-improved robustness against background fluorescence with the quantitative measurements. **d**, The curves of the mean intensities of the 27×27×42 μm^3 sub-volume reconstructed by different methods versus different mean background

intensities, demonstrating the quantitative property of QLFM. The dashed line illustrates the groundtruth for comparison. Scale bar, 10 μm.

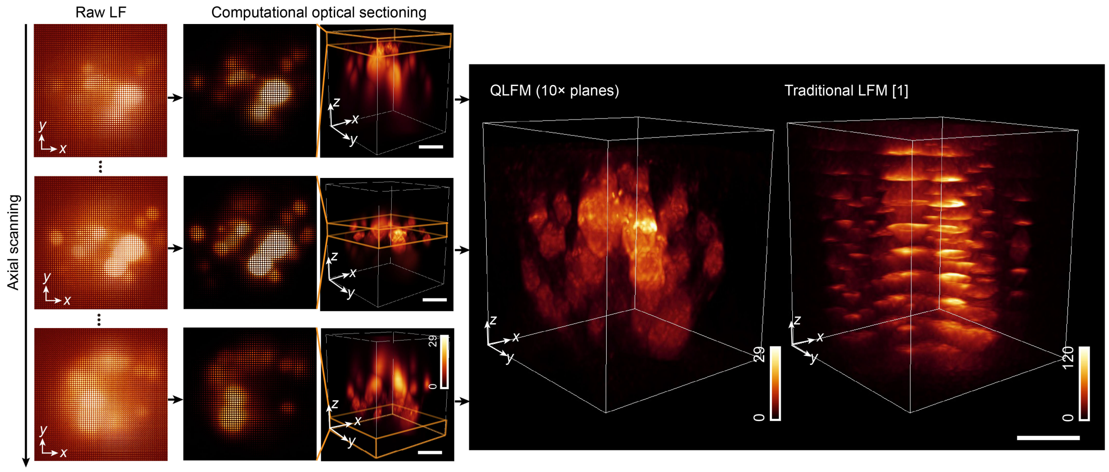

Supplementary Figure 4 | Large-scale volumetric imaging of GFP-labeled tumor spheroids with axial scanning. A GFP-labeled tumor spheroid was imaged at an axial step of 10 μm for 10 sub-volumes under a 63×/1.4 NA oil-immersion objective. Due to the capability of computational optical sectioning in QLFM, the large-scale volume was reconstructed as a whole with background and scattered photons removed, providing uniform resolution along the large axial range. In contrast, Strong edge artifact was observed on the 3D volume reconstructed by traditional methods due to the crosstalk from the background. Scale bar, 30 μm.

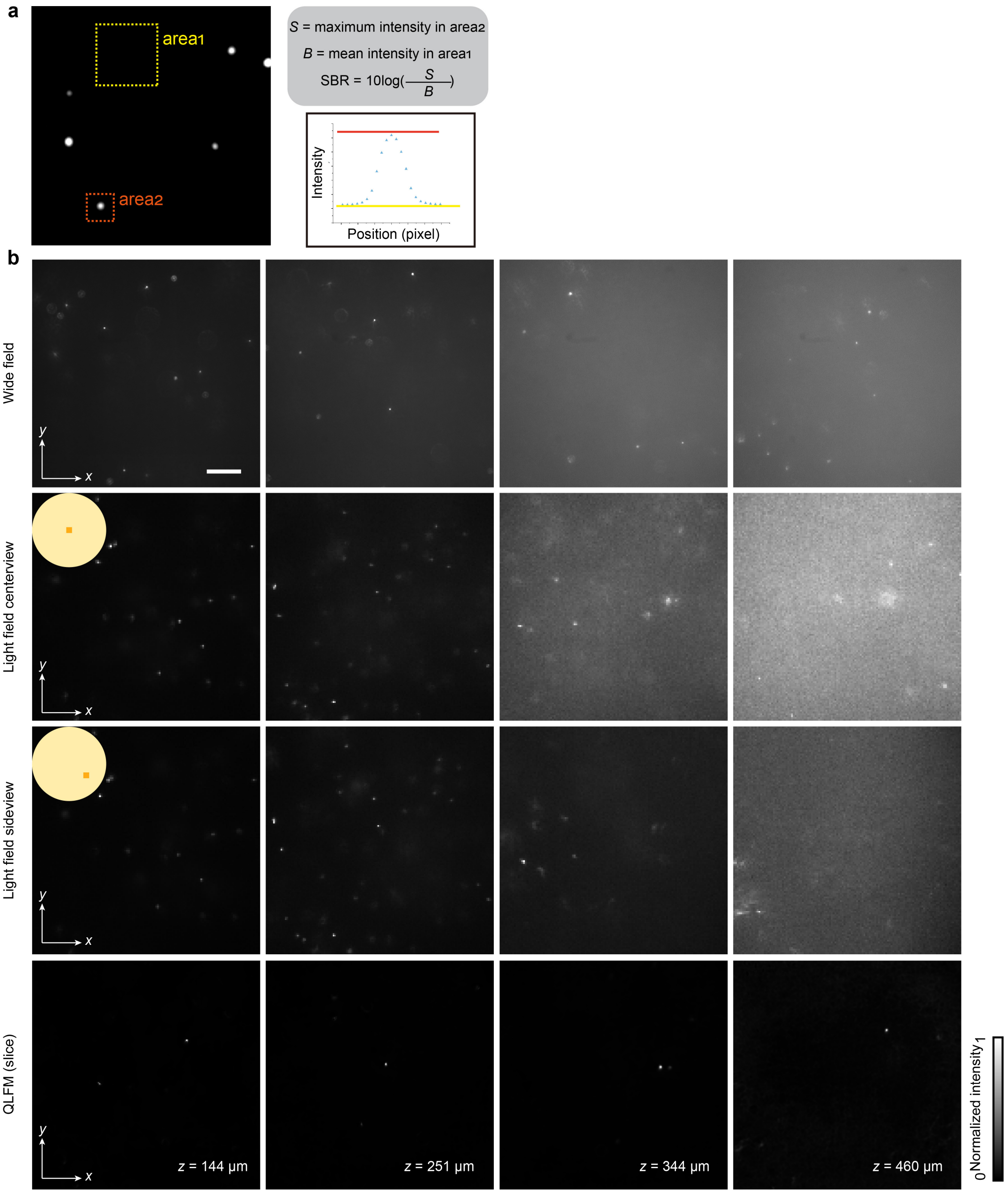

Supplementary Figure 5 | Characterization of the signal-to-background ratio (SBR) in the tissue-mimicking phantom. a, The SBR calculation. Average SBR is calculated by multiple fluorescence beads at the same penetration depth. **b**, We selected several typical areas at different penetration depths for comparisons among WFM, two angular views of LFM, and reconstructed slices by QLFM. QLFM show significantly improved SBR over WFM, illustrating a larger penetration depth in deep tissue with computational optical sectioning. Scale bar, 100 μm.

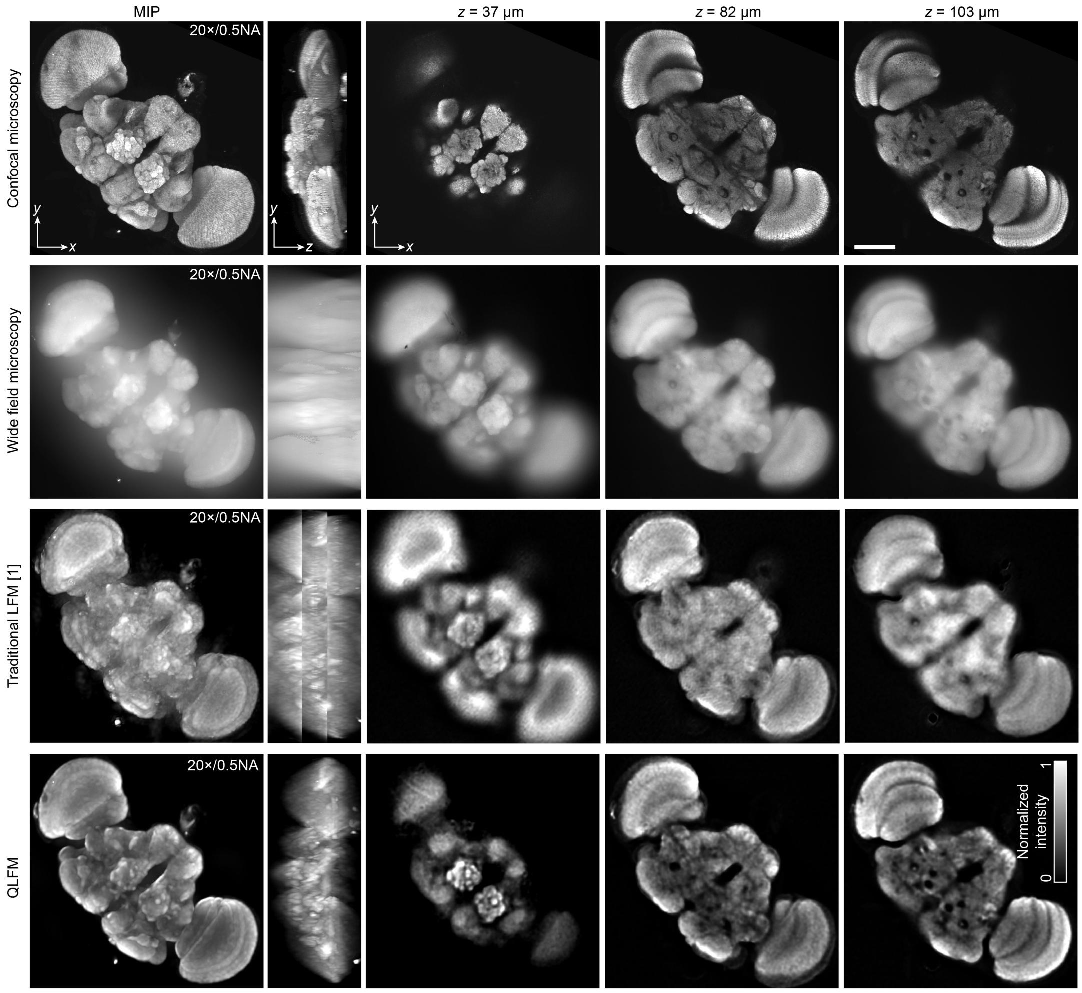

Supplementary Figure 6 | Experimental comparisons among confocal microscopy, WFM, traditional LFM, and QLFM on the same fixed *Drosophila* brain. The orthogonal maximum intensity projections (MIPs) and *xy*-slices obtained by different methods are shown on the different rows. During imaging, we used the same sample with a 20×/0.5NA objective. For confocal microscopy, we scanned the sample axially at a step of 1 μm for 139 slices. For WFM, we scanned 800 slices at 0.5-μm steps. For LFM, we scanned the entire volume with a step size of 50 μm by 3 times at 24 Hz, and reconstructed the volume by traditional LFM and QLFM, respectively. QLFM can not only get rid of the stitching artifacts with better contrast than traditional LFM, but also has a comparable optical-sectioning capability as confocal microscopy at a much faster 3D imaging speed. Scale bar, 100 μm.

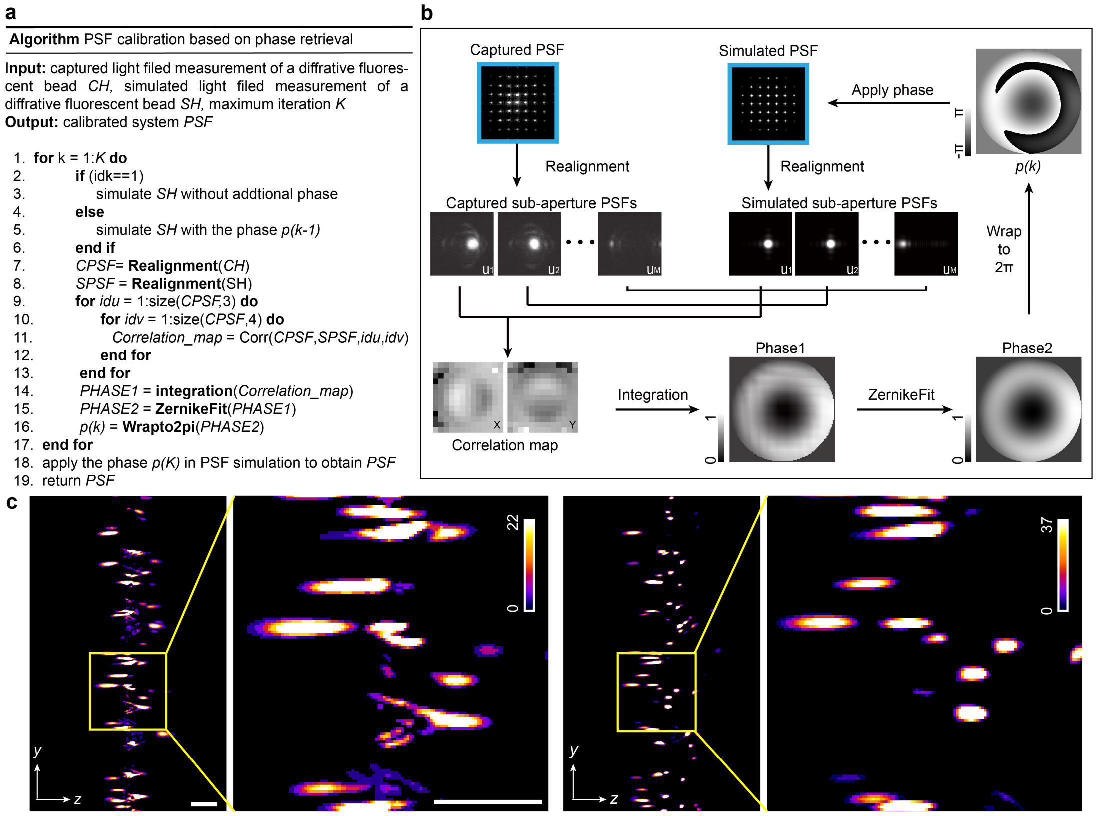

Supplementary Figure 7 | The pseudo codes and corresponding illustrations for phase-retrieval-based PSF calibration. **a**, Pseudo codes for the algorithm. **b**, Illustrations of the algorithm. As it's hard to capture the experimental PSF of LFM with a microlens array due to the high-dimensional property, simulated PSF based on wave optics is usually used during reconstruction. However, the system aberration of the experimental setup usually introduces severe system errors such as resolution degradation and artifacts. Here, we find that the accurate estimation of the PSF can eliminate the reconstruction artifacts close to the native objective plane in LFM and improve the high-resolution depth range. We used a single image of the sub-diffraction-limited fluorescence beads only, as marked by the blue box. Then, we generated the simulated LF PSF at a similar axial position. Both the captured and simulated images of a single point were realigned into phase-space domain in the form of multiple angular components, corresponding to different sub-apertures. Later, we calculated the disparities between the captured and simulated images along different angular components, which can be integrated as a phase map for calibration. After fitting with

Zernike polynomials, we apply the phase back to generate the PSF again for next iteration. The calibrated phase to estimate the system aberration will gradually converge after 5 iterations, which is then used for the calibrated PSF during reconstruction. **c**, Orthogonal MIPs of 0.5-μm fluorescence beads distributed in agarose reconstructed by QLFM with an ideal PSF without calibration (left) and a calibrated PSF (right), respectively. Scale bar, 20 μm.

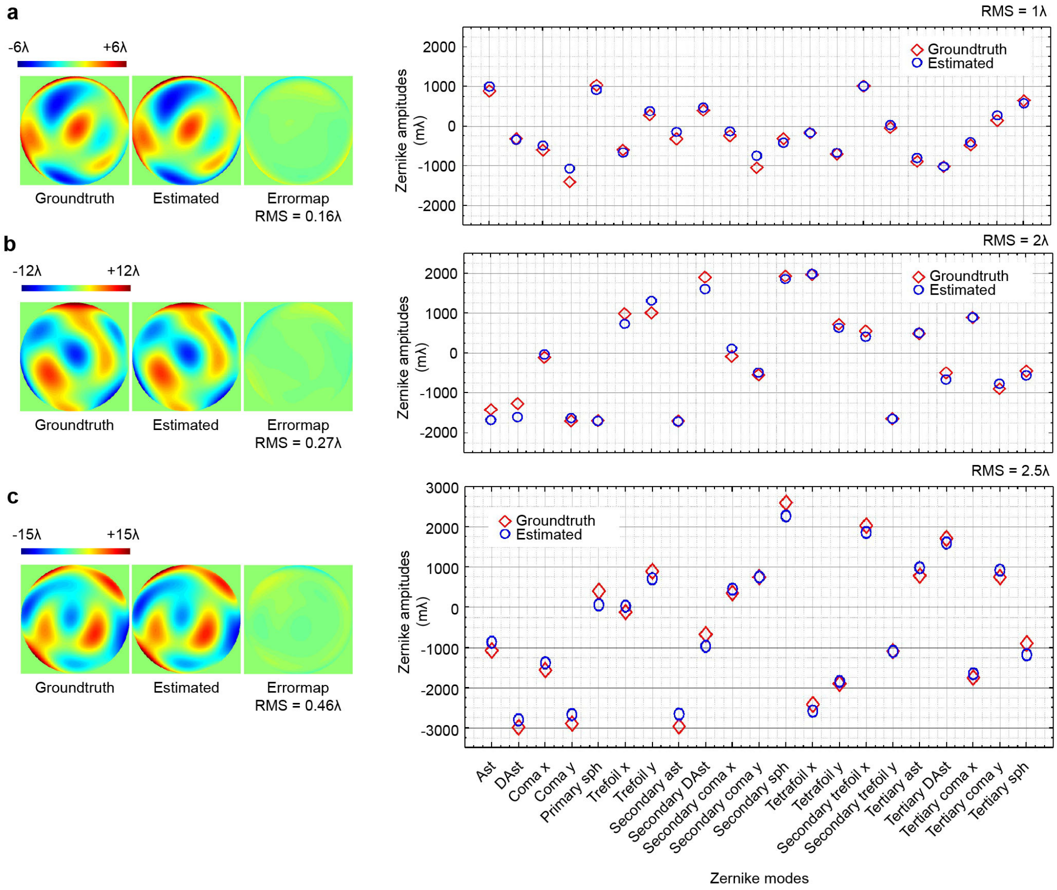

Supplementary Figure 8 | Quantitative analysis of the phase-retrieval-based PSF calibration algorithm. a-c, To evaluate the accuracy of the phase estimation, we conducted a numerical simulation with different levels of system aberrations applied to the system under a 20×/0.5NA objective. The groundtruth phase (left), the estimated phase by our algorithm (middle), and corresponding residual error map (right) are shown for comparisons. In addition, we visualized the amplitudes of 21 Zernike modes decomposed from the estimated pupils (blue circles) and the groundtruth (red diamonds), suggesting the effectiveness of the algorithm with a single image to calibrate the system. RMS, Root Mean Square.

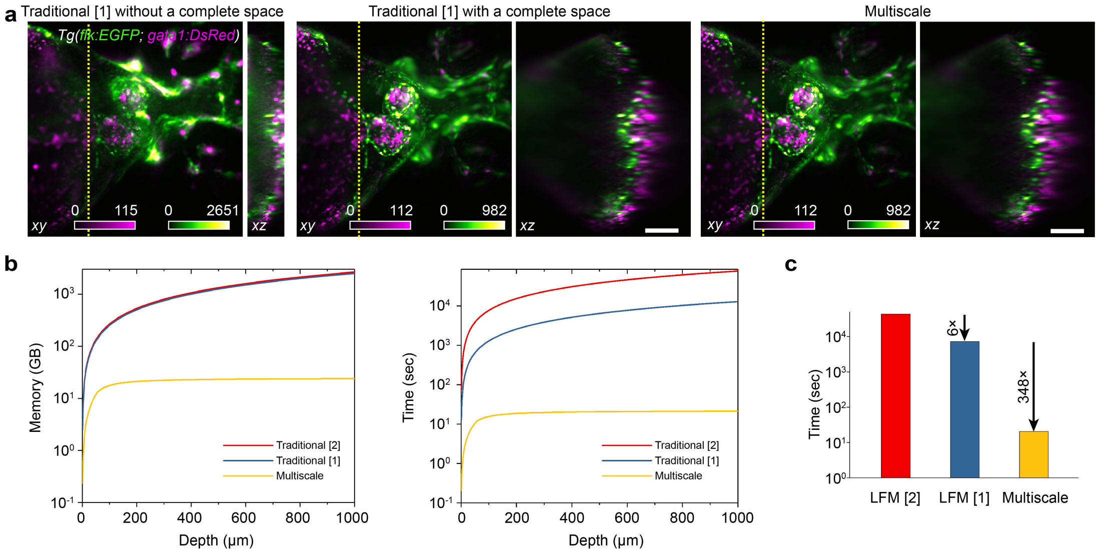

Supplementary Figure 9 | Illustrations of the effectiveness of multiscale model to reduce the memory and computing time by orders of magnitude. a, Orthogonal MIPs of the beating heart in zebrafish larvae reconstructed by different methods. The multiscale model shows similar performance as traditional methods in complete space with orders-of-magnitude reduction in computational costs. **b**, The curves of the required memory and computation time versus the reconstructed depth range for each volume by different methods with a 20×/0.5NA objective, indicating that the multiscale model is essential to cover a large depth of range for computational optical sectioning. **c**, The bar graph shows the time required by different methods to reconstruct the volumes with the same size covering about ~ 700×700×560 μm^3. We ran different methods on the same desktop computer (CPU: Intel i9-9820X, GPU: NVIDIA GeForce RTX 2080 Ti). Scale bar: 100 μm.

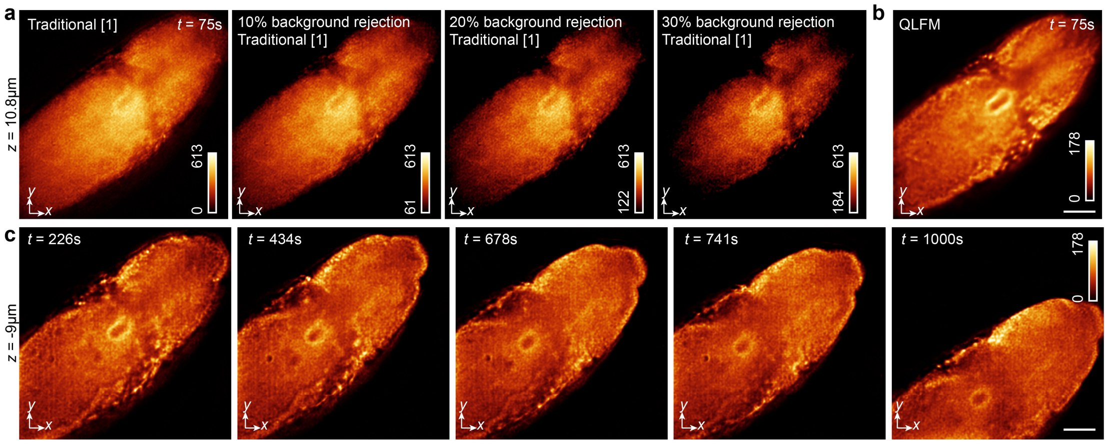

Supplementary Figure 10 | Experimental comparisons on a *Drosophila* embryo between traditional LFM and QLFM. a, Slices reconstructed by traditional method with different levels of background subtracted. **b**, The same slice reconstructed by QLFM at t = 75 s. Due to the removed background and scattered photons, QLFM offers single-cell resolution in thick scattering sample without any prior information. **c**, Slices of reconstructed *Drosophila* embryo at different time stamps. The morphological characteristics of the embryo and the positions of the cells change a lot during the developmental process, impeding traditional methods to resolve cells in thick samples without any prior. In contrast, QLFM is applicable in sustained morphological imaging across a long time with low phototoxicity. Scale bar: 50 μm.

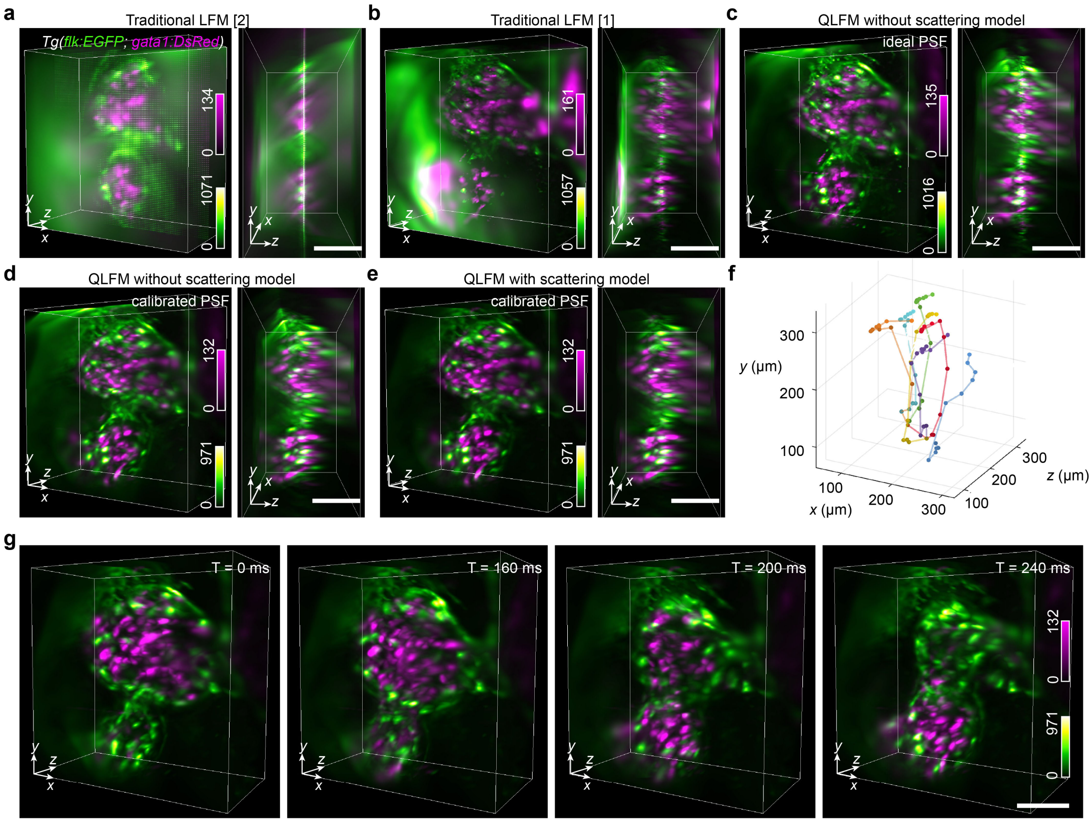

Supplementary Figure 11 | Experimental comparisons on the beating heart in zebrafish larvae to show improvements. a-e, The 3D rendered volumes reconstructed by traditional models[1,2], QLFM without the scattering model (using ideal PSF for reconstruction), QLFM without the scattering model (using calibrated PSF for reconstruction), and QLFM with the scattering model (using calibrated PSF for reconstruction), indicating the necessity of all the factors for quantitative volumetric reconstruction. The videos were captured with a 40×/1.0NA water-immersion objective at 25 Hz for two channels. All the methods are set to the same memory cost for fair comparisons. **f**, 3D tracking trajectories of representative blood cells flowing through the heart. **g**, 3D rendered volumes at different time stamps reconstructed by QLFM, showing engine-like dynamics of the heart-beating process. Scale bars, 50 μm

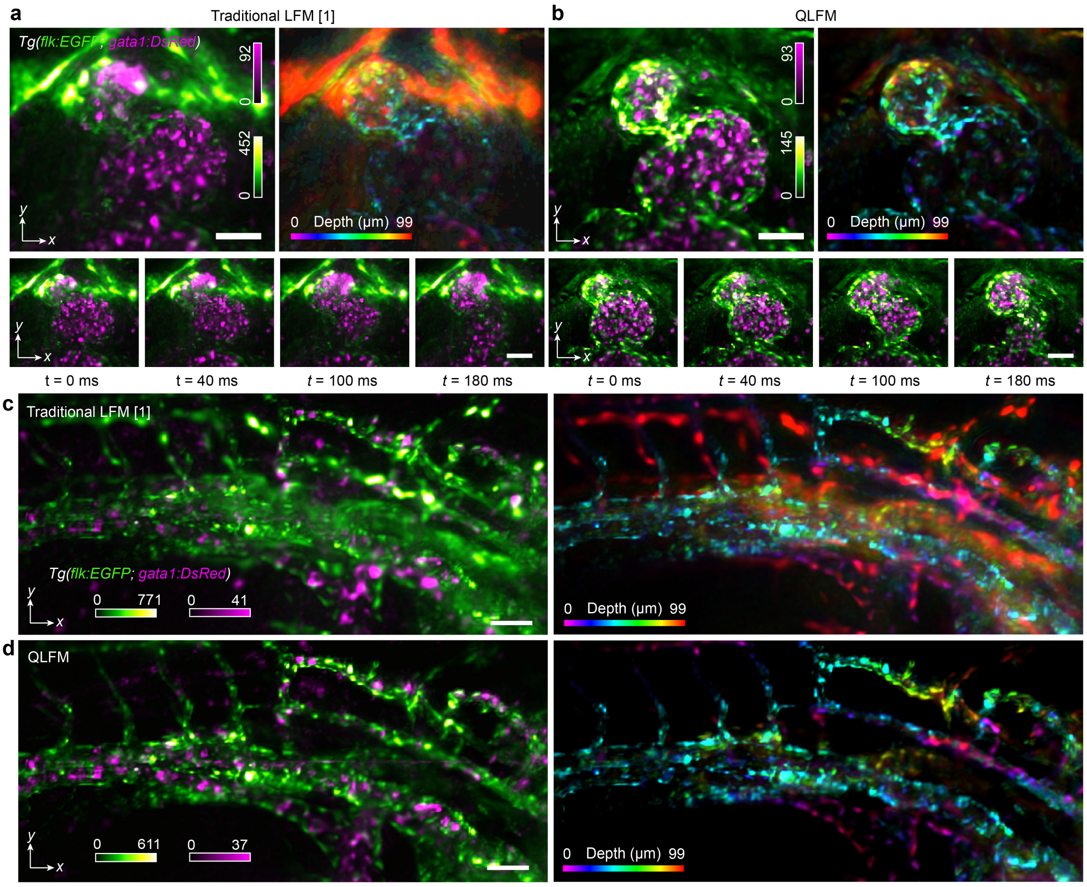

Supplementary Figure 12 | Experimental comparisons on the large-scale blood flow in zebrafish larvae between traditional LFM and QLFM. a-b, The two-color MIPs and depth-coded MIPs of a beating heart in zebrafish larvae reconstructed by traditional LFM and QLFM. The two-color MIPs at different time stamps are marked at the bottom row, illustrating the large-scale red blood cells (magenta) flowing in the vessels (green). For the depth-coded MIP, different colors correspond to different depths. **c-d**, The orthogonal MIPs and depth-coded MIPs for comparison in another region, demonstrating the application of large-scale 3D tracking. Scale bars, 50 μm.

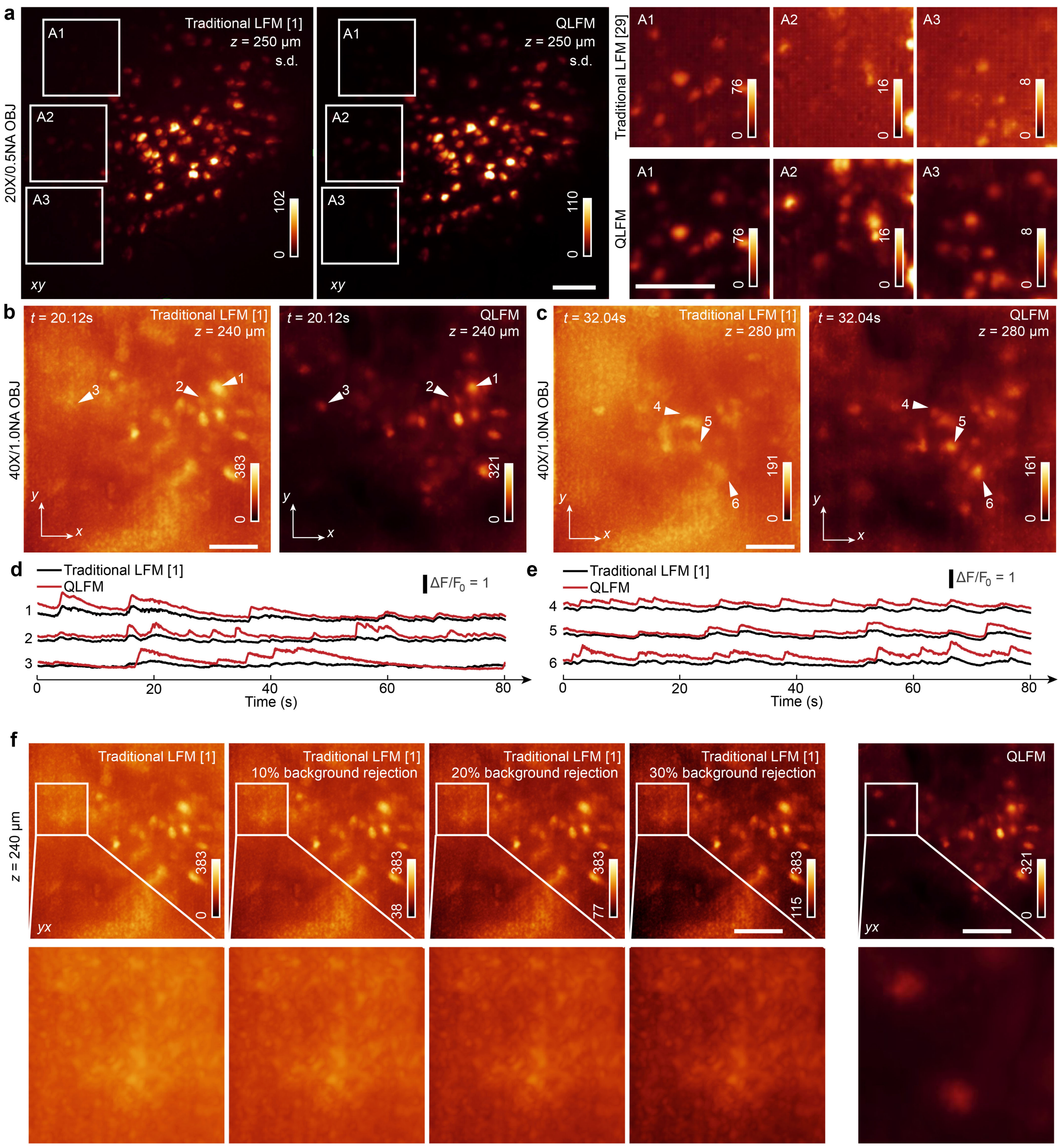

Supplementary Figure 13 | In vivo 3D calcium imaging in mouse cortex. a, The MIPs of the standard deviations (s.d.) for the 2000 volumes obtained by traditional LFM and QLFM under a 20×/0.5NA objective (Supplementary Movie 4). The MIPs are displayed at different contrast due to the high dynamic range of QLFM, indicating that QLFM can detect more neurons due to improved SBR. **b-c**, The comparisons of the MIPs at *t*=20.12s between traditional LFM and QLFM under a 40×/1.0NA water-immersion objective imaged at different depths, showing QLFM has larger penetration depth with computational optical sectioning. **d-e,** Temporal traces of marked neurons

in b and c, illustrating the better contrast. **f**, Orthogonal MIPs of volumes reconstructed by the QLFM and traditional method with different levels of direct background subtraction. The result indicates that QLFM resolves much more neurons, which cannot be obtained by simple background removal. Scale bars, 100 μm (**a**) and 50 μm (**b-c, f**).

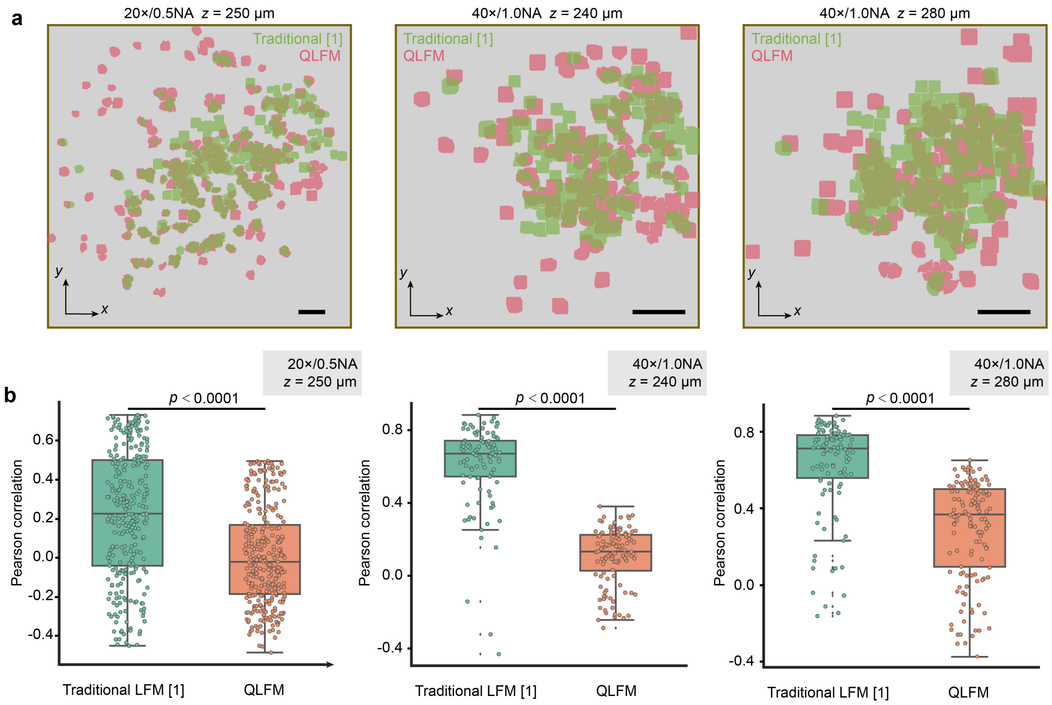

Supplementary Figure 14 | Comparisons on the performance of subsequent data analysis between traditional LFM and QLFM. a, CNMF segmentation results of neurons at various depth with 20×/0.5 NA and 40×/1.0 NA objectives. Each segmentation map is obtained by 1000 volumes reconstructed by traditional LFM (green) and QLFM (red)**,** respectively. **b,** QLFM reduced the correlation coefficients due to the removed background and scattered photons. Box-and-whisker plots show the Pearson correlation between the segmented neurons and the corresponding background under different imaging conditions: 20×/0.5 NA (depth = 250μm; Traditional LFM n= 287; QLFM n =317); 40×/1.0 NA (depth = 240μm; Traditional LFM, n=100; QLFM n =116); 40×/1.0 NA (depth = 280μm; Traditional LFM n= 106; QLFM n = 136). Experiments were examined over 6 independent experiments. The boxplot was plotted in the format: center line, median; box limits, 25th–75th percentiles; whiskers, 1.5× interquartile range (IQR). Data points outside of the whiskers were plotted as outliers. P values were determined via the unpaired, two-tailed t-test (α = 0.05). Source data are provided as a Source data file. Scale bars: 50 μm.

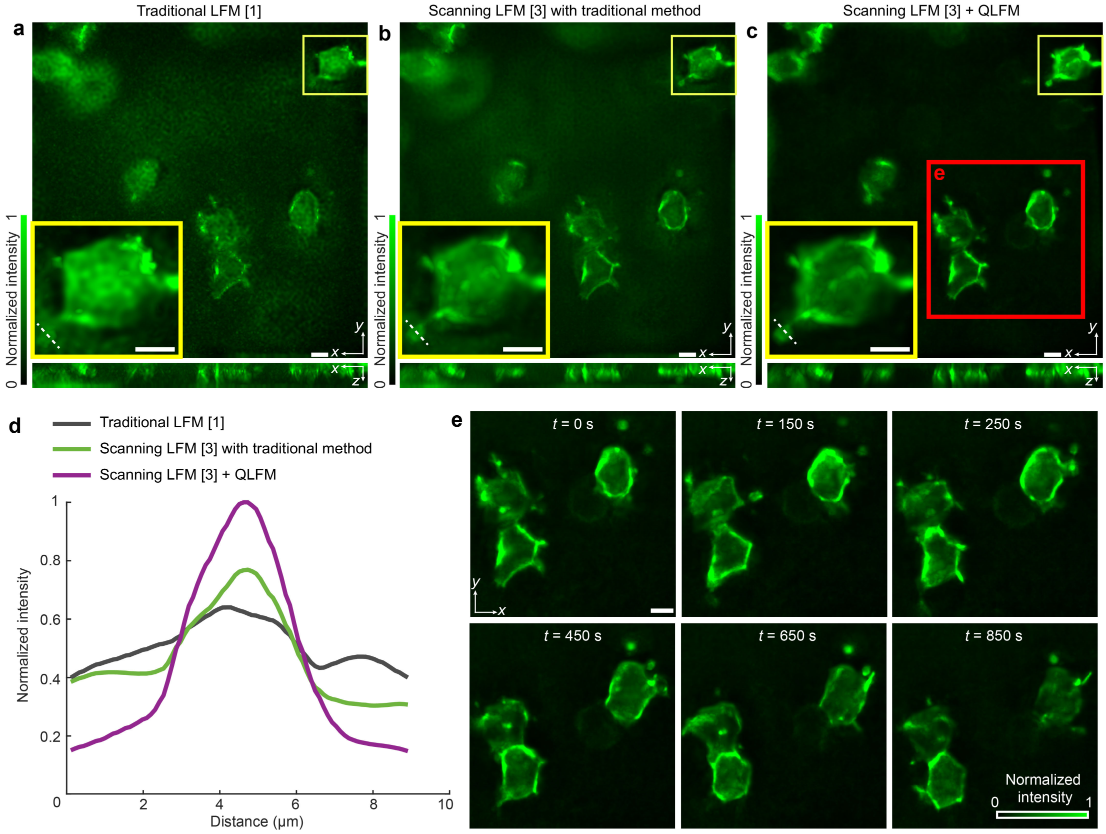

Supplementary Figure 15 | Experimental comparisons on the zebrafish embryo with scanning LFM. a, Orthogonal MIPs captured by LFM and reconstructed with the traditional model. Zebrafish embryos were injected with 300 pg *Tspan4a*-EGFP mRNA in one cell at 16-cell stage and the *Tspan4*-EGFP expressing embryo cells were imaged at 70% epiboly stage. **b-c,** Orthogonal MIPs captured by scanning LFM[3], reconstructed with the traditional model and multiscale scattering model, indicating the valid background reduction in scanning LFM setup. **d,** The profiles of the marked white dash lines in a-c, showing contrast enhancement with multiscale scattering model. **e,** The MIPs of the marked red box in c at different time stamps captured by scanning LFM and reconstructed with multiscale scattering model, showing subcellular embryo dynamics. Scale bars, 10 μm.

Supplementary Notes

Supplementary Note 1. Incoherent scattering model for 3D deconvolution.

Considering a monochromatic electromagnetic field (the time-dependent factor is not shown), the scattering medium is limited to a finite large area V modeled in a multiscale way, and satisfies linearity, isotropy and non-magnetic property. The space-dependent part of the complex electric field $\mathbf{E}$ can be expressed by:

$$\nabla^2\mathbf{E}(\mathbf{r},\omega)+k^2\varepsilon(\mathbf{r},\omega)\mathbf{E}(\mathbf{r},\omega)+\mathrm{grad}[\mathbf{E}(\mathbf{r},\omega)\cdot\mathrm{grad}\ln\varepsilon(\mathbf{r},\omega)]=0\ , \qquad (1)$$

where $\mathbf{r}=(x,y,z)$ is the 3D spatial positions, ω is the frequency coordinate, $k=\omega/c$ is the wave number, and ∇^2 is the Laplace operator. Then, we assume the dielectric coefficient $\varepsilon(\mathbf{r},\omega)$ is approximately constant in the range of center wavelength $\lambda=2\pi/k=2\pi(c/\omega)$ with the emission filter. So, Eq. (1) can be simplified as

$$\nabla^2\mathbf{E}(\mathbf{r},\omega)+k^2n^2(\mathbf{r},\omega)\mathbf{E}(\mathbf{r},\omega)=0\ , \qquad (2)$$

where $n(\mathbf{r},\omega)$ is the medium refractive index, satisfying Maxwell's equation $\varepsilon(\mathbf{r},\omega)=n^2(\mathbf{r},\omega)$. The scalar form of Eq. (2) is written as:

$$\nabla^2\mathbf{U}(\mathbf{r},\omega)+k^2n^2(\mathbf{r},\omega)\mathbf{U}(\mathbf{r},\omega)=0\ . \qquad (3)$$

The Eq. (3) is tranformed into

$$\begin{gathered}\nabla^2U(\mathbf{r},\omega)+k^2U(\mathbf{r},\omega)=-4\pi F_0(\mathbf{r},\omega)U(\mathbf{r},\omega)\\ F_0(\mathbf{r},\omega)=\frac{1}{4\pi}k^2\left[n^2(\mathbf{r},\omega)-1\right]\end{gathered}\ , \qquad (4)$$

where $F_0(\mathbf{r},\omega)$ is defined as the scattering potential energy. The field $U(\mathbf{r},\omega)$ represents the sum of incident field $U^{(i)}(\mathbf{r},\omega)$ and scattering field $U^{(s)}(\mathbf{r},\omega)$:

$$U(\mathbf{r},\omega)=U^{(i)}(\mathbf{r},\omega)+U^{(s)}(\mathbf{r},\omega)\ . \qquad (5)$$

The incident field usually can be regarded as a plane wave, which satisfies Helmholtz equation:

$$\left(\nabla^2 + k^2\right)U^{(i)}(\mathbf{r},\omega) = 0 \cdot \tag{6}$$

The intensity field can be obtained as:

$$I(\mathbf{r},\omega) = U(\mathbf{r},\omega)\cdot U^*(\mathbf{r},\omega). \tag{7}$$

By substituting Eq.(5) into Eq.(7) and ignoring the crossed terms, the intensity field is written as:

$$I(\mathbf{r},\omega) = I^{(i)}(\mathbf{r},\omega) + I^{(s)}(\mathbf{r},\omega), \tag{8}$$

where $I^{(i)}(\mathbf{r},\omega)$ is the incident intensity and $I^{(s)}(\mathbf{r},\omega)$ is the scattering intensity. The second order derivative of the intensity field $I(\mathbf{r},\omega)$ is then developed into:

$$\begin{aligned}\nabla^2 I(\mathbf{r},\omega) &= \nabla^2(U(\mathbf{r},\omega)\cdot U^*(\mathbf{r},\omega)) \\ &= U(\mathbf{r},\omega)\nabla^2 U^*(\mathbf{r},\omega) + U^*(\mathbf{r},\omega)\nabla^2 U(\mathbf{r},\omega)\end{aligned}. \tag{9}$$

Using Eq. (6), Eq. (9) can be tranformed into:

$$\nabla^2 I(\mathbf{r},\omega) + 2k^2 I(\mathbf{r},\omega) = -4\pi F(\mathbf{r},\omega) I(\mathbf{r},\omega), \tag{10}$$

where

$$F(\mathbf{r},\omega) = 2F_0(\mathbf{r},\omega) = \frac{1}{2\pi}k^2\left[n^2(\mathbf{r},\omega) - 1\right]. \tag{11}$$

Using Eq. (6) and Eq. (8), Eq. (10) can be tranformed into:

$$(\nabla^2 + 2k^2) I^{(s)}(\mathbf{r},\omega) = -4\pi F(\mathbf{r},\omega) I(\mathbf{r},\omega). \tag{12}$$

Then, we convert the differential equation to an integral equation. We let $G(\mathbf{r}-\mathbf{r}')$ be the Green function of Helmholtz operator, which satisfies

$$\left(\nabla^2 + k^2\right)G\left(\mathbf{r}-\mathbf{r}',\omega\right) = -4\pi\delta^{(3)}\left(\mathbf{r}-\mathbf{r}'\right), \tag{13}$$

where $\delta^{(3)}(\cdot)$ is the 3D Dirac impulse function. We take the conjugate of Eq. (13) to obtain

$$\left(\nabla^2 + k^2\right)G^*\left(\mathbf{r}-\mathbf{r}',\omega\right) = -4\pi\delta^{(3)}\left(\mathbf{r}-\mathbf{r}'\right)\cdot \tag{14}$$

Eq. (13) and Eq. (14) can be combined as:

$$\left(\nabla^2 + 2k^2\right)\left\|G\left(\mathbf{r}-\mathbf{r}',\omega\right)\right\|_2^2 = -4\pi\delta^{(3)}\left(\mathbf{r}-\mathbf{r}'\right)\left[G\left(\mathbf{r}-\mathbf{r}',\omega\right) + G^*\left(\mathbf{r}-\mathbf{r}',\omega\right)\right]\cdot \tag{15}$$

Then with Eq. (11), we can obtain:

$$
\begin{aligned}
& I^{(s)}(\mathbf{r},\omega)\nabla^2 \left\| G\left(\mathbf{r}-\mathbf{r}',\omega\right)\right\|_2^2 - \left\| G\left(\mathbf{r}-\mathbf{r}',\omega\right)\right\|_2^2 \nabla^2 I^{(s)}(\mathbf{r},\omega) \\
&= 4\pi F(\mathbf{r},\omega) I(\mathbf{r},\omega) \left\| G\left(\mathbf{r}-\mathbf{r}',\omega\right)\right\|_2^2 \\
&\quad - 4\pi I^{(s)}(\mathbf{r},\omega)\delta^{(3)}\left(\mathbf{r}-\mathbf{r}'\right)\left[G\left(\mathbf{r}-\mathbf{r}',\omega\right)+G^*\left(\mathbf{r}-\mathbf{r}',\omega\right)\right]
\end{aligned} . \tag{16}
$$

By converting volume integration to surface integration, we have

$$
\begin{aligned}
& I^{(s)}(\mathbf{r},\omega) \\
&= \frac{1}{2}\int_V F\left(\mathbf{r}',\omega\right) I\left(\mathbf{r}',\omega\right)\left\| G\left(\mathbf{r}-\mathbf{r}',\omega\right)\right\|_2^2 \mathrm{d}^3 r' \\
&= -\frac{1}{8\pi}\int_{S_R}\left[I^{(s)}\left(\mathbf{r}',\omega\right)\frac{\partial \left\| G\left(\mathbf{r}-\mathbf{r}',\omega\right)\right\|_2^2}{\partial n'} - \left\| G\left(\mathbf{r}-\mathbf{r}',\omega\right)\right\|_2^2 \frac{\partial I^{(s)}\left(\mathbf{r}',\omega\right)}{\partial n'}\right]\mathrm{d}S_R
\end{aligned} , \tag{17}
$$

where V is the range of volume integration, and the spherical surface S_R surrounds the scattering area[4]. Here, we select the Green function as below (from now on we leave out the frequency ω):

$$
G\left(\mathbf{r}-\mathbf{r}'\right) = \frac{\mathrm{e}^{\mathrm{i}k\left|\mathbf{r}-\mathbf{r}'\right|}}{\left|\mathbf{r}-\mathbf{r}'\right|}, \tag{18}
$$

and

$$
\left\| G\left(\mathbf{r}-\mathbf{r}'\right)\right\|_2^2 = \frac{1}{\left\|\mathbf{r}-\mathbf{r}'\right\|_2^2}. \tag{19}
$$

In the far-field range of the scattering volume, the scattered field can often be assumed to have the characteristics of a spherical wave. Taking the limit $R \to \infty$, the surface integral on the right side of Eq. (17) has no contribution to the total field[6,7]. Therefore, the scattered field is simplified as:

$$
I^{(s)}(\mathbf{r}) = \frac{1}{2}\int_V F\left(\mathbf{r}'\right) I\left(\mathbf{r}'\right)\frac{1}{\left\|\mathbf{r}-\mathbf{r}'\right\|_2^2}\mathrm{d}^3 r'. \tag{20}
$$

For simplicity, the total field in Eq. (20) is approximated as the incident intensity based on the assumption of weak scattering, leading to the expression as below:

$$
I^{(s)}(\mathbf{r}) = \frac{1}{2}\int_V F\left(\mathbf{r}'\right) I^{(i)}\left(\mathbf{r}'\right)\frac{1}{\left\|\mathbf{r}-\mathbf{r}'\right\|_2^2}\mathrm{d}^3 r'. \tag{21}
$$

where $\mathbf{r}$ can be written as $\mathbf{r}=(\boldsymbol{\rho},z)$, $\boldsymbol{\rho}=(x,y)$ is the lateral coordinate, and z is the axial coordinate.

For the discrete model in reconstruction, the 3D object is divided into multiple slices. Each slice has a thin but finite thickness Δz. The relation between the incident intensity and the scattering intensity in n^{th} slice can be written as:

$$\begin{aligned} I^{(s)}(\boldsymbol{\rho},n\Delta z) &= \frac{1}{2}\Delta z\int_S F\left(\boldsymbol{\rho}',n\Delta z\right) I^{(i)}\left(\boldsymbol{\rho}',n\Delta z\right)\frac{1}{\left\|\boldsymbol{\rho}-\boldsymbol{\rho}'\right\|_2^2}\mathrm{d}\boldsymbol{\rho}' \\ &= C\left[F\left(\boldsymbol{\rho},n\Delta z\right) I^{(i)}\left(\boldsymbol{\rho},n\Delta z\right)\right]\otimes\frac{1}{\left\|\boldsymbol{\rho}\right\|_2^2} \\ &= C\left[F\left(\boldsymbol{\rho},n\Delta z\right) I^{(i)}\left(\boldsymbol{\rho},n\Delta z\right)\right]\otimes\delta\left(\boldsymbol{\rho}\right) \\ &= CF\left(\boldsymbol{\rho},n\Delta z\right) I^{(i)}\left(\boldsymbol{\rho},n\Delta z\right) \end{aligned}, \tag{22}$$

where $\delta(\cdot)$ is the 2D Dirac impulse function, C is a constant, omitted for simplicity, S is the range of one slice, and $\otimes$ represents the convolution operator. Considering forward and backward scattering processes, the incident intensity in n^{th} slice can be divided as:

$$I^{(i)}\left(\boldsymbol{\rho},n\Delta z\right) = I_F^{(i)}\left(\boldsymbol{\rho},n\Delta z\right) + I_B^{(i)}\left(\boldsymbol{\rho},n\Delta z\right), \tag{23}$$

where $I_F^{(i)}\left(\boldsymbol{\rho},n\Delta z\right)$ represents the forward incident intensity in n^{th} slice and $I_B^{(i)}\left(\boldsymbol{\rho},n\Delta z\right)$ represents the backward incident intensity. These formulas can be written as:

$$\begin{aligned} I_F^{(i)}\left(\boldsymbol{\rho},n\Delta z\right) &= \left[I_F^{(i)}\left(\boldsymbol{\rho},(n-1)\Delta z\right) + X\left(\boldsymbol{\rho},(n-1)\Delta z\right)\right]\otimes\frac{1}{\left\|\mathbf{r}\right\|_2^2} \\ I_B^{(i)}\left(\boldsymbol{\rho},n\Delta z\right) &= \left[I_B^{(i)}\left(\boldsymbol{\rho},(n+1)\Delta z\right) + X\left(\boldsymbol{\rho},(n+1)\Delta z\right)\right]\otimes\frac{1}{\left\|\mathbf{r}\right\|_2^2}, \\ I_B^{(i)}\left(\boldsymbol{\rho},N\Delta z\right) &= 0 \\ I_F^{(i)}\left(\boldsymbol{\rho},0\right) &= 0 \end{aligned} \tag{24}$$

where $X\left(\boldsymbol{\rho},n\Delta z\right)$ represents the emitted fluorescence intensity in n^{th} slice and N is the total number of the slices. The Eq. (22) can be developed into

$$\begin{aligned} I^{(s)}(\boldsymbol{\rho},n\Delta z) &= F(\boldsymbol{\rho},n\Delta z) I^{(i)}(\boldsymbol{\rho},n\Delta z) \\ &= F(\boldsymbol{\rho},n\Delta z)\cdot\left[I_F^{(i)}(\boldsymbol{\rho},n\Delta z)+I_B^{(i)}(\boldsymbol{\rho},n\Delta z)\right]. \end{aligned} \tag{25}$$

To estimate the scattering intensity and the fluorescence intensity, we consider $I^{(s)}(\mathbf{r},\omega)$ in the 3D deconvolution algorithm with ADMM framework[5], as shown in Supplementary Fig. 2. The whole 3D volume and PSF are sampled according to the designed multiscale sampling rate. Within each iteration, the phase-space deconvolution is used to update the reconstructed volume $X_{all}(\boldsymbol{\rho},n\Delta z)$, which consists of the native fluorescence intensity $X(\boldsymbol{\rho},n\Delta z)$ and the scattering intensity $I^{(s)}(\boldsymbol{\rho},n\Delta z)$:

$$X_{all}(\boldsymbol{\rho},n\Delta z) = X(\boldsymbol{\rho},n\Delta z) + I^{(s)}(\boldsymbol{\rho},n\Delta z). \tag{26}$$

The $I^{(s)}(\boldsymbol{\rho},n\Delta z)$ and $X(\boldsymbol{\rho},n\Delta z)$ can then be obtained directly from the fixed scattering potential $F(\boldsymbol{\rho},n\Delta z)$ based on Eq. (25). $F(\boldsymbol{\rho},n\Delta z)$ is initialized to zero for the first iteration.

Then, we fix the sample intensity $X(\boldsymbol{\rho},n\Delta z)$ to update the scattering potential $F(\boldsymbol{\rho},n\Delta z)$ with the gradient descent method to solve the following optimization problem:

$$\min_F L$$

$$\begin{aligned} L &= \left\|\sum_n H_u(\boldsymbol{\rho},n\Delta z)\otimes X_{all}(\boldsymbol{\rho},n\Delta z) - W_u(\boldsymbol{\rho})\right\| \\ &= \left\|\sum_n H_u(\boldsymbol{\rho},n\Delta z)\otimes\left\{X(\boldsymbol{\rho},n\Delta z)+F(\boldsymbol{\rho},n\Delta z)I^{(i)}(\boldsymbol{\rho},n\Delta z)\right\} - W_u(\boldsymbol{\rho})\right\|_2^2 \end{aligned}, \tag{27}$$

$$\text{s.t. } 0 \le F(\boldsymbol{\rho},n\Delta z) \le 1$$

where $H_u(\boldsymbol{\rho},n\Delta z)$ is the PSF for a specific angle u, and $W_u(\boldsymbol{\rho})$ is the captured angular component along angle u after pixel realignemnt. Then the update formula for the scattering potential $F(\boldsymbol{\rho},n\Delta z)$ can be written as :

$$F\left(\boldsymbol{\rho}, n\Delta z\right) = F\left(\boldsymbol{\rho}, n\Delta z\right) - \alpha \frac{\partial L}{\partial F}, \tag{28}$$

where $\frac{\partial L}{\partial F} = 2\left(\sum_n H_u\left(\boldsymbol{\rho}, n\Delta z\right) \otimes X_{all}\left(\boldsymbol{\rho}, n\Delta z\right) - W_\omega\left(\boldsymbol{\rho}\right)\right) \otimes H_u{}^T\left(-\boldsymbol{\rho}, n\Delta z\right) \cdot I^{(i)}\left(\boldsymbol{\rho}, n\Delta z\right)$, $H_u{}^T\left(-\boldsymbol{\rho}, n\Delta z\right)$ is the transpose PSF for a specific angle and α is the learning rate. Then, the updated scattering potential $F\left(\boldsymbol{\rho}, n\Delta z\right)$ is fed again to the next iteration for another angle. We repeat the above ADMM steps along every angular components iteratively for convergence. Each angular component is used for only one time during iterations for all the experiments without axial scanning, while each angular component is used for two times for all the experiments with axial scanning.

References

1. Lu, Z. *et al.* Phase-space deconvolution for light field microscopy. *Opt. Express* **27**, 18131-18145 (2019).
2. Prevedel, R. *et al.* Simultaneous whole-animal 3D imaging of neuronal activity using light-field microscopy. *Nat. Methods* **11**, 727-730 (2014).
3. Wu, J. *et al.* Iterative tomography with digital adaptive optics permits hour-long intravital observation of 3D subcellular dynamics at millisecond scale. *Cell* (2021). https://doi.org/10.1016/j.cell.2021.04.029.
4. Born, M., Wolf, E. & Hecht, E. Principles of Optics: Electromagnetic Theory of Propagation, Interference and Diffraction of Light. *Phys. Today* (2000).
5. Boyd, Stephen, Neal Parikh, Eric Chu, Borja Peleato, and Jonathan Eckstein. "Distributed Optimization and Statistical Learning via the Alternating Direction Method of Multipliers." *Foundations and Trends in Machine Learning*. (2010).
6. Colton, David, and Rainer Kress. Inverse Acoustic and Electromagnetic Scattering Theory: Fourth Edition. *Applied Mathematical Sciences*. (2019).
7. Sneddon, I. N., B. B. Baker, and E. T. Copson. "The Mathematical Theory of Huygens' Principle." *The Mathematical Gazette*. (1951).

Supplementary Table 1. Imaging conditions for all fluorescence experiments.

	Sample, (imaging T, ℃)	Fluorescent label	Exposure time (# time pts)	λ: Power (mW/mm^2)	Volume rate (Hz)	Objective	Axially-scanned planes and spacing
1d	Tumor spheroid 27 °C	GFP	100 ms 1 pts	488: 1.21		63×/1.4NA Oil	1 plane
1c，S4, SM1	Tumor spheroid 27 °C	GFP	100 ms 1 pts	488: 1.21	-	63×/1.4NA Oil	10 planes 10 µm
2b, S5	Tissue-mimicking phantom 27 °C	Yellow-green fluorescent (505/515)	20 ms 800 pts	488: 11.7	-	40×/1.0NA Water	1 plane
2c	Fluorescence beads 27 °C	Yellow-green fluorescent (505/515)	95 ms 1 pts	488: 11.7	-	40×/1.0NA Water	1 plane
24, S7c	Fluorescence beads 27 °C	Yellow-green fluorescent (505/515)	100 ms 100 pts	488: 11.7	-	40×/1.0NA Water	5 planes 30 µm
3a-d, S10	*Drosophila* embryos 25℃	EGFP (His2Av)	95 ms 1 pts	488: 11.7	-	40×/1.0NA Water	8 planes 15 µm
3e-f	*Drosophila* embryos 25℃	EGFP (His2Av)	300 ms 1600 pts	488: 7.1	1	20×/0.5NA Air	3 planes 50 µm
4a-c, S11, SM2	Zebrafish larval 27℃	*Tg(flk:EGFP; gata1:DsRed)*	10 ms 100 pts	488: 61.9 561:80.5	25	40×/1.0NA Water	1 plane
4d-f, SM3	Zebrafish larval 27℃	*Tg(huc:GCamp6)*	30 ms 4000 pts	488: 12.5	24	20×/0.5NA Air	1 plane

5a-b, S13a SM4	Awake behaving mouse 27℃	GCaMP6s	30 ms 2000 pts	488:3.6	25	20×/0.5NA Air	1 plane
5e-f, S13b-c, f, SM5	Awake behaving mouse 27℃	GCaMP6s	30 ms 2000 pts	488:3.4	25	40×/1.0NA Water	1 plane
5g, SM6	Awake behaving mouse 27℃	GCaMP6f	30 ms 500 pts	488: 1.4	6	20×/0.5NA Air	3 planes 50 µm
S6	*Drosophila* brain 25℃	Antibody nc82	100 ms 1 pts	488: 11.0	-	20×/0.5NA Air	5 planes 50 µm
S12	Zebrafish larval 27°C	*Tg(flk:EGFP; gata1:DsRed)*	10 ms 50 pts	488: 5.0 561: 9.0	25	20×/0.5NA Air	1 plane
S15a-c, e	Zebrafish embryo 27°C	EGFP	50 ms 1300 pts	488: 3.9	10hz	63x/1.4NA Oil	1 plane

Supplementary Table 2. Rendering parameters and models for all fluorescence experiments.

Figure #	software	colormap	Display mode
1d	Amira	Glow	Voltex (MIP), Slice
2c	Matlab	Cyan & magenta	Slice
2e	Matlab	Fire	MIP
3a,b	Amira	Glow	Voltex(*xz*-MIP) &Volren (Volume rendering)
3c	Matlab	Spectrum	Depth
3d	Matlab	Glow	Slice
3e-f	Amira	Glow	Voltex(*xz*-MIP) &Volren (Volume rendering)
4a-c	Amira	Greenhot & magenta	Voltex (MIP)
4d-g, h	Amira	Greenhot	Voltex (MIP), Slice
5a-b, e-f, g	Amira	Glow	Voltex (MIP)
S4	Amira	Glow	Voltex (MIP), Slice
S5	Matlab	Grays	Slice
S6	Matlab	Grays	Slice
S7c	Matlab	Fire	MIP
S9a	Matlab	Greenhot & magenta	MIP
S10	Matlab	Glow	Slice
S11	Matlab	Greenhot & magenta	MIP
S12	Matlab	Greenhot & magenta	MIP
S12	Matlab	Spectrum	Depth
S13a-c, f	Amira	Glow	Voltex (MIP)
S15a-c, e	Matlab	Green	MIP
