# Supplementary material for: Computational optical sectioning with an incoherent multiscale scattering model for light-field microscopy
Source: Nat Commun. 2021 Nov 4;12:6391. doi: 10.1038/s41467-021-26730-w (PMC8568979; doi:10.1038/s41467-021-26730-w)
Supplement: Supplementary file 2 — Description of Additional Supplementary Files [file 41467_2021_26730_MOESM2_ESM.docx]

**Description of Additional Supplementary Files:**

**Supplementary Movie 1** | Concept and pipeline of the multiscale scattering model for 3D deconvolution. The first part of the video shows the comparison of depth of focus between Wide field and LFM. The fluorescence signals shown in different angular components extracted from LF data have distinguishable features. The second part of the video shows the 3D deconvolution algorithm with multiscale scattering model. The last part of the video shows the volumetric reconstruction result of a GFP labelled tumour spheroid. Strong edge artefact was observed in the volume reconstructed by traditional method, while QLFM provides a better estimation of the background and uniform resolution along the z axial.

**Supplementary Movie 2** | Heart-beating dynamics in zebrafish larvae imaged at 25 Hz by two channels to show the improvements with the model complexity. The video shows 3D renderings (Amira5.4 Volren) of the volumes reconstructed by traditional models, QLFM without the scattering model (using ideal PSF for reconstruction), QLFM without the scattering model (using calibrated PSF for reconstruction), and QLFM with the scattering model (using calibrated PSF for reconstruction). With more factors taken into consideration, the model performs better.

**Supplementary Movie 3** | Experimental comparisons on whole brain calcium imaging of zebrafish larvae at 24Hz. The movie shows the comparison of volumetric renderings reconstructed by traditional methods and QLFM. QLFM shows single-neuron resolution and reduces the cross-talk between adjacent neurons by eliminating both out of-focus and scattered photons.

**Supplementary Movie 4** | Experimental comparisons on 3D calcium imaging of a virus-injected awake mouse with a 20×/0.5 NA objective. While calcium signals were flooded by the background fluctuations in the volumes reconstructed by traditional method, QLFM significantly reduced the cross-talks with better SBR.

**Supplementary Movie 5** | Experimental comparisons on 3D calcium imaging of a virus-injected awake mouse with a 40×/1.0 NA water-immersion objective. We imaged the same mouse at different depth. The video shows that QLFM can resolve more neurons with better penetration depth and better SBR for downstream analysis.

**Supplementary Movie 6** | Experimental comparisons on 3D calcium imaging of a transgenic awake mouse with high-speed axial scanning. We imaged awake double-transgenic Rasgrf2-2AdCre/Ai148D mice by high-speed axial scanning of 3 planes. Strong edge artefacts and background can be observed in traditional methods, while QLFM resolves more neurons with better contrast for a large depth range that can be flexibly adjusted
